# ‘What’ and ‘How’ to Measure in Allergy and Clinical Immunology: A Systematic Review of Core Outcome Sets and Outcome Harmonisation Processes

**DOI:** 10.1111/cea.70251

**Published:** 2026-04-13

**Authors:** Anastasia Demidova, Nata Kiknavelidze, Kristine Purtskhvanidze, Elvina Alieva, Mehrshad Ebrahimnejad, Svetlana Konchina, Azaliya Nurmeeva, Igor Matkovskii, Elmira Elmurzaeva, Siuzanna Davtian, Natalia Degtyareva, Karl Philipp Drewitz, Alan Asmanov, Nikolina Banjanin, Erna Botjes, Pasquale Comberiati, Joana Costa, Derek K. Chu, Michelle M. Epstein, Lyudmila Fedorova, Audrey Dunn Galvin, Mattia Giovannini, Matthew Greenhawt, Kristina R. Jamalyan, Christina J. Jones, Ekaterina Khaleva, Rebecca C. Knibb, Yael A. Leshem, Douglas P. Mack, Isabel Mafra, Mary Jane Marchisotto, Dragan Mijakoski, Asel Nurtazina, Cevdet Özdemir, Diego Peroni, Jennifer L. P. Protudjer, Pablo Rodríguez del Río, Ann‐Marie Malby Schoos, Anita Fossaluzza Schopfer, Sasho Stoleski, Julia Upton, Willem van de Veen, Jon Genuneit, Robert J. Boyle, Christian Apfelbacher, Daniel Munblit

**Affiliations:** ^1^ Independent Researcher Limassol Cyprus; ^2^ Tbilisi State Medical University Faculty of Medicine Tbilisi Georgia; ^3^ Department of Paediatrics and Paediatric Infectious Diseases, Institute of Child's Health, I.M. Sechenov First Moscow State Medical University Sechenov University Moscow Russia; ^4^ Institute of Social Medicine and Health Systems Research Otto von Guericke University, Medical Faculty Magdeburg Germany; ^5^ Veltischev Scientific Research Clinical Institute of Pediatrics and Children Surgery Pirogov Russian National Research Medical University Moscow Russia; ^6^ Institute of Hygiene and Medical Ecology, Faculty of Medicine University of Belgrade Belgrade Serbia; ^7^ Dutch Food Allergy SVA the Netherlands; ^8^ Department of Clinical and Experimental Medicine, Section of Paediatrics University of Pisa Pisa Italy; ^9^ REQUIMTE‐LAQV, Faculdade de Farmácia Universidade Do Porto Porto Portugal; ^10^ Department of Medicine, and Department of Health Research Methods, Evidence, and Impact McMaster University Hamilton Ontario Canada; ^11^ Experimental Allergy Laboratory, Department of Dermatology Medical University of Vienna Vienna Austria; ^12^ School of Applied Psychology University College Cork Cork Ireland; ^13^ Allergy Unit Meyer Children's Hospital IRCCS Florence Italy; ^14^ Department of Health Sciences University of Florence Florence Italy; ^15^ Section of Allergy and Immunology Children's Hospital Colorado Aurora Colorado USA; ^16^ Department of Pediatrics University of Colorado School of Medicine Aurora Colorado USA; ^17^ Department of Immunology and Allergology National Institute of Health Republic of Armenia; ^18^ School of Psychology University of Surrey Guildford UK; ^19^ Human Development and Health, Faculty of Medicine University of Southampton Southampton UK; ^20^ Aston Institute of Health and Neurodevelopment Aston University Birmingham UK; ^21^ Faculty of Medicine & Health Sciences Tel Aviv University Tel Aviv Israel; ^22^ Division of Dermatology Rabin Medical Center Petah‐Tikva Israel; ^23^ Department of Pediatrics McMaster University Hamilton Ontario Canada; ^24^ MJM Advisory LLC New York New York USA; ^25^ Institute of Occupational Health of R.N. Macedonia WHO Collaborating Center Skopje North Macedonia; ^26^ Faculty of Medicine, Ss. Cyril and Methodius University in Skopje Skopje North Macedonia; ^27^ Department of Clinical Immunology and Allergology I.M. Sechenov First Moscow State Medical University, Sechenov University Moscow Russia; ^28^ Institute of Child Health, Department of Pediatric Basic Sciences Istanbul University Istanbul Türkiye; ^29^ Department of Pediatrics and Child Health, Max Rady College of Medicine, Rady Faculty of Health Sciences University of Manitoba Winnipeg Manitoba Canada; ^30^ Children's Hospital Research Institute of Manitoba Winnipeg Manitoba Canada; ^31^ Department of Food and Human Nutritional Sciences, Faculty of Agricultural and Food Sciences University of Manitoba Winnipeg Manitoba Canada; ^32^ Institute of Environmental Medicine Karolinska Institutet Stockholm Sweden; ^33^ Allergy Department Hospital Infantil Universitario Niño Jesús Madrid Spain; ^34^ FibHNJ RICORS Center RD24/0007/0037 Instituto de Salud Carlos III Madrid Spain; ^35^ IIS La Princesa Madrid Spain; ^36^ COPSAC, Copenhagen Prospective Studies on Asthma in Childhood Copenhagen University Hospital—Herlev and Gentofte Gentofte Denmark; ^37^ Department of Pediatrics Copenhagen University Hospital—Næstved Slagelse Denmark; ^38^ Department of Pediatrics Copenhagen University Hospital—Amager and Hvidovre Hvidovre Denmark; ^39^ Allergissima Association Switzerland; ^40^ The Hospital for Sick Children, Divison of Immunology and Allergy, Department of Paediatrics, Faculty of Medicine University of Toronto Toronto Canada; ^41^ Swiss Institute of Allergy and Asthma Research (SIAF) University of Zurich Davos Switzerland; ^42^ Pediatric Epidemiology, Department of Pediatrics, Medical Faculty Leipzig University Leipzig Germany; ^43^ National Heart and Lung Institute Imperial College London London UK; ^44^ Care for Long Term Conditions Division, Florence Nightingale Faculty of Nursing, Midwifery and Palliative Care King's College London London UK

**Keywords:** allergic diseases, clinical trial, consensus, core outcome set, Delphi, harmonisation, immunological conditions, measurement instrument, outcome assessment, quality of life, systematic review

## Abstract

**Background:**

Heterogeneity in outcome reporting and inconsistent use of outcome measurement instruments in allergy and clinical immunology research affects the comparability, synthesis, and clinical applicability of study findings. Harmonisation efforts, particularly Core Outcome Set (COS) development, aim to address these challenges by establishing standardised, evidence‐based and consensus‐driven outcome recommendations. This systematic review aims to map available COS and other harmonisation processes (HP) in allergy and clinical immunology, evaluate their methodological approaches, and assess their alignment with established development standards.

**Methods:**

We systematically searched MEDLINE, EMBASE, and the COMET Initiative database until June 7, 2024 to identify COS and HP. We included studies if they provided recommendations on ‘core’ outcomes and/or outcome measurement instruments. Data extraction included disease focus, methodological approach, stakeholder involvement, and adherence to the Core Outcome Set‐STAndards for Development criteria. We synthesised the data at the initiative (process) level rather than the publication level because harmonisation initiatives are frequently iterative and reported across multiple papers (e.g., protocol, Delphi rounds, consensus statement, and subsequent instrument‐selection outputs).

**Results:**

A total of 15,612 records were identified, with 44 studies (representing 22 initiatives both finished and in development) meeting inclusion criteria. The majority of initiatives focused on asthma (*n* = 9), followed by eczema (atopic dermatitis *n* = 2; hand eczema = 1; eczema = 1), urticaria (*n* = 2), allergic rhinitis (*n* = 2), chronic rhinosinusitis (*n* = 1), celiac disease (*n* = 1), Immunoglobulin E (IgE)—mediated food allergy (*n* = 1), eosinophilic esophagitis (*n* = 1), and hereditary angioedema (*n* = 1). No COS or HP addressed drug allergy, anaphylaxis, or other immune‐mediated allergic conditions. ‘Quality of life’ was consistently included in all COS with ‘signs and symptoms’, ‘exacerbations’ and ‘disease control’ frequently selected as well. Methodological approaches to COS development varied widely, with most employing Delphi surveys, consensus meetings, and stakeholder involvement, though levels of engagement differed. COS developers inconsistently adhered to Core Outcome Set‐STAndards for Development criteria, with some initiatives demonstrating rigorous methodology while others lacked transparency in key developmental steps.

**Conclusion:**

This review highlights growing efforts to harmonise outcome assessment in allergy and clinical immunology. Major gaps remain in coverage and methodological rigour. Quality of life and patient‐reported symptoms are frequently recommended outcomes, yet definitions and measurement tools are inconsistent. Strengthening methodological consistency and expanding COS development to neglected areas are critical next steps to improve outcome reliability and comparability in the field.

## Key Messages

- Core Outcome Sets (COS) exist for asthma, eczema, and food allergy but not for anaphylaxis or immunological conditions.
- Quality of life and symptoms were most commonly recommended outcomes across COS and harmonisation processes.
- Methodological rigour and stakeholder involvement in outcome harmonisation initiatives remain inconsistent across development processes.

## Graphical abstract

This systematic review maps core outcome sets and harmonisation efforts in allergy and clinical immunology. Despite shared priorities such as quality of life and symptoms, substantial gaps and methodological inconsistencies remain, underscoring the need for broader coverage and standardised outcome measurement to improve comparability and clinical relevance of future research.

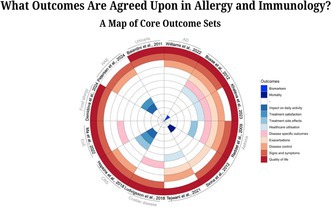

**Abbreviations**
ACQ‐6: asthma control questionnaire (6‐item version)
ACT: asthma control test
ADCT: atopic dermatitis control tool
C‐ACT: childhood asthma control test
COMS: core outcome measurement set
COMSA: core outcome measurement set for asthma
COREOS: core outcome set for eosinophilic oesophagitis
COS: core outcome set
COS‐STAD: Core Outcome Set‐STAndards for Development
CU‐Q2oL: chronic Urticaria questionnaire on quality of Life
DLQI: Dermatology Life Quality Index
DSQ: dysphagia symptoms questionnaire
EAACI: European Academy of Allergy and Clinical Immunology
EASI: Eczema Area and Severity Index
EEsAI: Eosinophilic Esophagitis Activity Index
EoE‐QoL‐A: adult eosinophilic oesophagitis quality of life
EREFS: eosinophilic esophagitis endoscopic reference score
FEV1: forced expiratory volume in one second
FVC: forced vital capacity
GA^2^LEN: Global Allergy and Asthma European Network
HAE: hereditary angioedema
HOME: harmonising outcome measures for eczema
HP: harmonisation processes
IgE: Immunoglobulin E
LABA: long‐acting beta agonist
NRS‐11: numeric rating scale (11‐point version)
OMI: outcome measurement instruments
PAQLQ: paediatric asthma quality of life questionnaire
PedsQL: paediatric quality of life inventory
PEESS: Paediatric Eosinophilic Esophagitis Symptom Score
PERN: paediatric emergency research networks
POEM: patient‐oriented eczema measure
PRISMA: preferred reporting items for systematic reviews and meta‐analyses
SABA: short‐acting beta agonist
SAQ: standardised asthma quality of life questionnaire
TAI: test of adherence to inhalers
UAS: Urticaria Activity Score
WPAI: work productivity and activity impairment questionnaire

## 1 Introduction

Heterogeneous outcome reporting and the inconsistent use of outcome measurement instruments (OMI) in clinical research across the fields challenge the reliability, comparability, and synthesis of study findings [1]. In clinical trials and other research contexts, the absence of standardised outcomes can lead to selective reporting, serve as a barrier to meta‐analyses and the translation of research results into clinical practice as well as contribute to research waste [2].

The rapid evolution of research and clinical practice in allergy and clinical immunology resulted in substantial heterogeneity in the outcomes and OMI used [3]. Outcome harmonisation initiatives sit on a continuum. A core outcome set (COS), recognised as the ‘gold standard’ [4], specifies what outcome domains should be measured and reported, as a minimum, for a given condition, population, and setting [5]. A core outcome measurement set (COMS) complements a COS by specifying how to measure those outcomes, including the preferred measurement instruments, definitions, timing and recall periods, where relevant. We use the term harmonisation processes (HP) for consensus‐based initiatives that recommend standard outcomes and/or measurement instruments but are not explicitly framed as COS/COMS (e.g., endpoint standardisation statements, taskforce recommendations, or registry outcome harmonisation frameworks).

Despite the increasing number of COS and other harmonisation activities for different allergic/immunological conditions such as asthma [6, 7, 8, 9, 10, 11, 12, 13, 14], eczema [15, 16, 17, 18], and food allergy [19, 20], there remains considerable variability in the methodologies employed and in the extent to which these processes adhere to established development standards, such as the Core Outcome Set‐STAndards for Development (COS‐STAD) [5]. While harmonisation efforts are important, uptake of COS into research remains suboptimal [21] influenced by both development‐stage factors—such as stakeholder involvement and ease of implementation—and broader external elements like regulatory support and community awareness.

Although COS are increasing in allergy and clinical immunology, their development for each disease condition is currently haphazard and opportunistic and therefore risks the same inconsistency that justified the need for COS in the first place. A systematic review of harmonisation processes in allergy and clinical immunology might inform targeted and systematic COS development. Unlike prior reviews that focused on single diseases, this review provides a field‐wide mapping across allergy and selected clinical immunology conditions and includes COS, COMS and other harmonisation initiatives, synthesised at the initiative level with COS‐STAD appraisal. Selected immunological conditions were included because they are commonly managed within allergy/immunology services and share methodological challenges with allergic diseases, including rarity, phenotypic heterogeneity and the need for patient‐reported and registry‐based outcomes. We therefore systematically reviewed outcomes and measurement instruments from harmonisation processes conducted in the field of allergy and clinical immunology.

## 2 Methods

This systematic review is reported in accordance with the recommendations set forth by the Preferred Reporting Items for Systematic Reviews and Meta‐Analyses (PRISMA) statement. The review was registered with the National Institute for Health Research's PROSPERO 2024 CRD42024564904. Available from: https://www.crd.york.ac.uk/prospero/display_record.php?ID=CRD42024564904 on Jul 10, 2024.

### 2.1 Search Strategy

A systematic electronic search of MEDLINE and EMBASE databases via OVID covered a period from inception to June 7, 2024, using both free‐text and MeSH terms. An additional search was conducted via the Core Outcome Measures in Effectiveness Trials (COMET) initiative registry (https://comet‐initiative.org/) with the final search updated on March 4, 2025. The detailed search strategies are available in Table S1.

### 2.2 Eligibility Criteria

#### 2.2.1 Conditions of Interest

Any allergic condition or primary immunodeficiency was eligible for inclusion. This includes COS related to all allergy conditions as per the 2023 Novel Classification of Allergic Disorders [22]. For studies pertaining to immunological conditions, we drew upon the 2017 IUIS Phenotypic Classification for Primary Immunodeficiencies [23], specifically focusing on Groups I, II, and III. Immunological conditions focusing on groups IV, V, VI, VII, VIII, IX from the 2017 IUIS Phenotypic Classification for Primary Immunodeficiencies were excluded from analysis.

All COS/core outcome measurement set (COMS)/outcome harmonisation consensus processes were eligible, regardless of whether they focused on a specific demographic (e.g., adults or children) or targeted a particular intervention or exposure (e.g., drug, medical device).

#### 2.2.2 Outcomes of Interest

‘Core’ (most critical) outcomes, outcome measurement instruments/definitions as defined within the reviewed COS/COMS/HP for the allergic and immunological conditions recommended for research purposes (including clinical trials) and clinical practice.

#### 2.2.3 Types of Studies

Any outcome harmonisation efforts were included when they (1) addressed diseases in allergy and clinical immunology; (2) provided recommendations regarding outcomes/endpoints and/or outcome measurement instruments/definitions; and (3) published in English. Studies were included regardless of the methodology used and intended settings (e.g., clinical trials, research, clinical practice, etc.). Studies were excluded if they focused solely on patients' perspectives of outcomes as per earlier similar evidence syntheses [24], reported on outcome usage without developing a core set, involved animals, reported the use of an existing COS, were based on a single author's opinion, or if they were clinical trials, observational studies, letters, conference abstracts, editorials, or commentaries.

#### 2.2.4 Types of Participants

All COS/COMS, HP studies were included unless they solely involved patient perspective.

### 2.3 Study Selection and Data Extraction

Articles identified in EMBASE and MEDLINE databases were transferred into Covidence systematic review software, Veritas Health Innovation, Melbourne, Australia. To reduce potential selection bias, pairs of authors (N.K., K.P., E.A., S.K., N.D., M.E.) independently screened the same titles and abstracts of the identified studies and reviewed all the studies for suitability for full‐text assessment. Articles and protocols identified in the COMET database were also independently screened for suitability. Any disagreements between the screeners were resolved via consensus or team discussion with the involvement of at least two additional reviewers (A.D., D.Mu). Data extraction was also conducted independently by the same pairs of authors. Extracted data included the condition of interest, type of process, intended setting, intervention and population, demographics of the study participants, methodology used for the outcome/OMI selection, process results, conflict of interest handling, and funding source.

### 2.4 Study Definitions and Mapping

In this study, we define the ‘COS’ as one in which authors clearly outline their aim to develop a core set of outcomes. ‘COMS’ is defined when authors recommend OMI for ‘core outcomes’ assessment. The ‘HP’ was defined as an effort to align outcome/OMI collection practices and provide recommendations on which outcomes should be measured in specific settings. HP included, for example, endpoint standardisation statements, taskforce position papers and registry outcome harmonisation frameworks when they provided explicit recommendations on a minimum set of outcomes and/or measurement instruments for a defined condition/setting. We did not include routine clinical practice guidelines that simply recommend endpoints within guideline development; guideline‐adjacent documents were included only if they reported an explicit outcome harmonisation exercise (e.g., consensus‐based standardisation of endpoints for trials/registries/clinical practice).

COS and harmonisation processes were mapped over the European Academy of Allergy and Clinical Immunology (EAACI) nomenclature of allergic diseases and hypersensitivity reactions [22].

### 2.5 COS‐STAD Assessment

Pairs of authors (N.K., K.P., E.A., S.K., N.D., M.E.) independently evaluated each study against the COS‐STAD guidelines. This assessment included 12 criteria representing the 11 minimum standards (Tables S2, S3). Each criterion was rated as ‘yes’ (meeting the standard), ‘no’ (not meeting the standard), or ‘unsure’ (uncertain if the standard was met). All assessments were then discussed with the team and a third additional reviewer (A.D.). Initiatives in development were mapped descriptively but were not used to draw conclusions about methodological rigour. COS‐STAD ratings were based on available reporting; when information was not reported, items were rated as ‘unclear’.

## 3 Results

### 3.1 Synthesis

**FIGURE 1:**
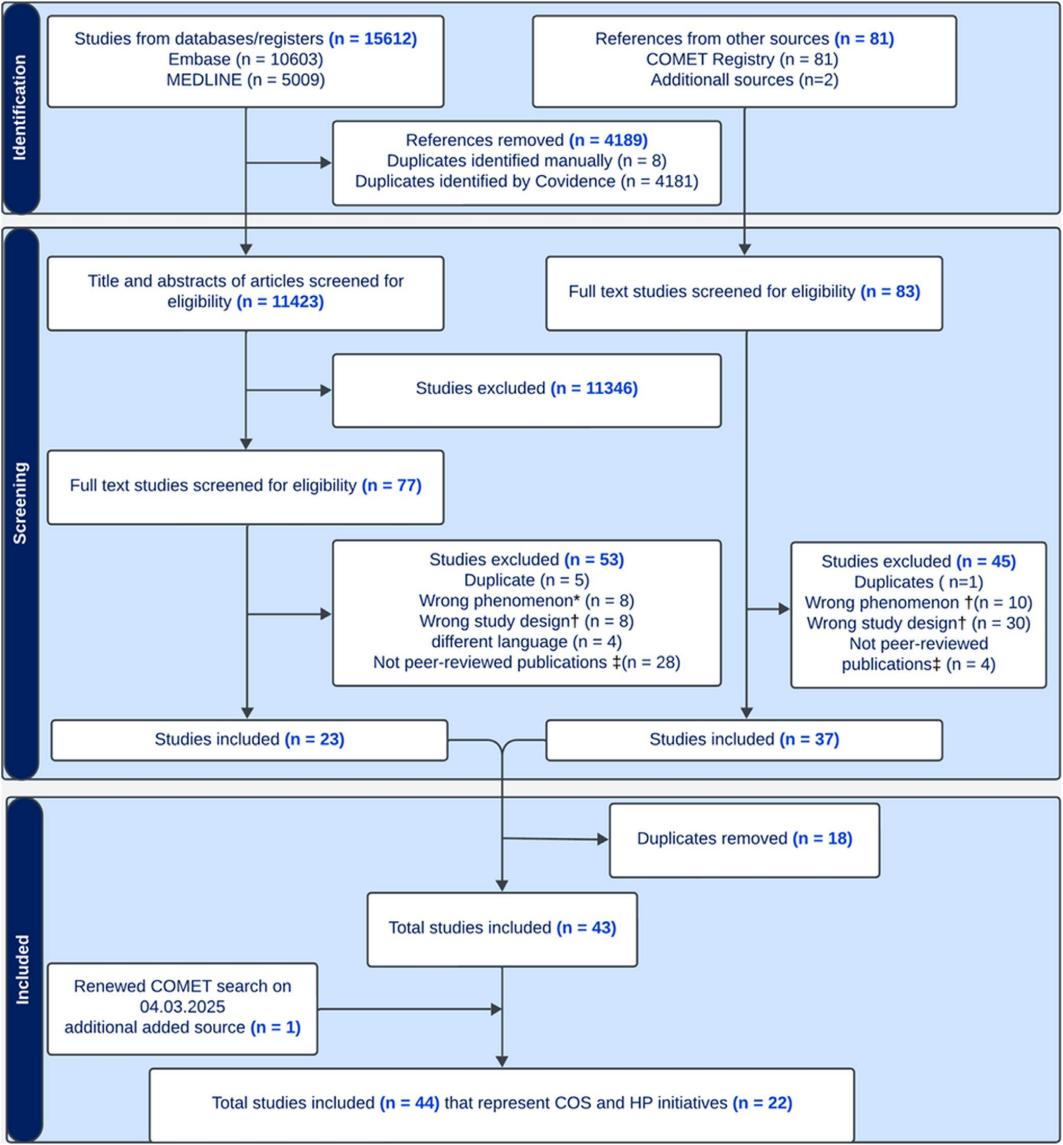
PRISMA flow chart. *Wrong phenomenon includes studies focused on the development of core datasets for registries only, studies on COS uptake/implementation, and studies on mixed conditions. †Wrong design includes review papers, clinical trials, and observational studies; ‡ Non‐peer‐reviewed publications include conference abstracts, letter/correspondence, news/notes, and opinion articles.

A total of 15,612 items were identified from searching MEDLINE and EMBASE databases. After removing duplicates, 11,423 published records remained (Figure 1). Based on title and abstract screening, 11,346 publications were excluded. A full‐text review was conducted for 77 published studies and 83 studies identified through other sources (81 from the COMET database via initial search and two via discussions with experts in the field). One study was added as a result of an updated search. As a result, our systematic review included 44 unique papers, representing results from 14 completed initiatives with eight initiatives currently under development (Table S4).

### 3.2 Included Initiatives

**FIGURE 2:**
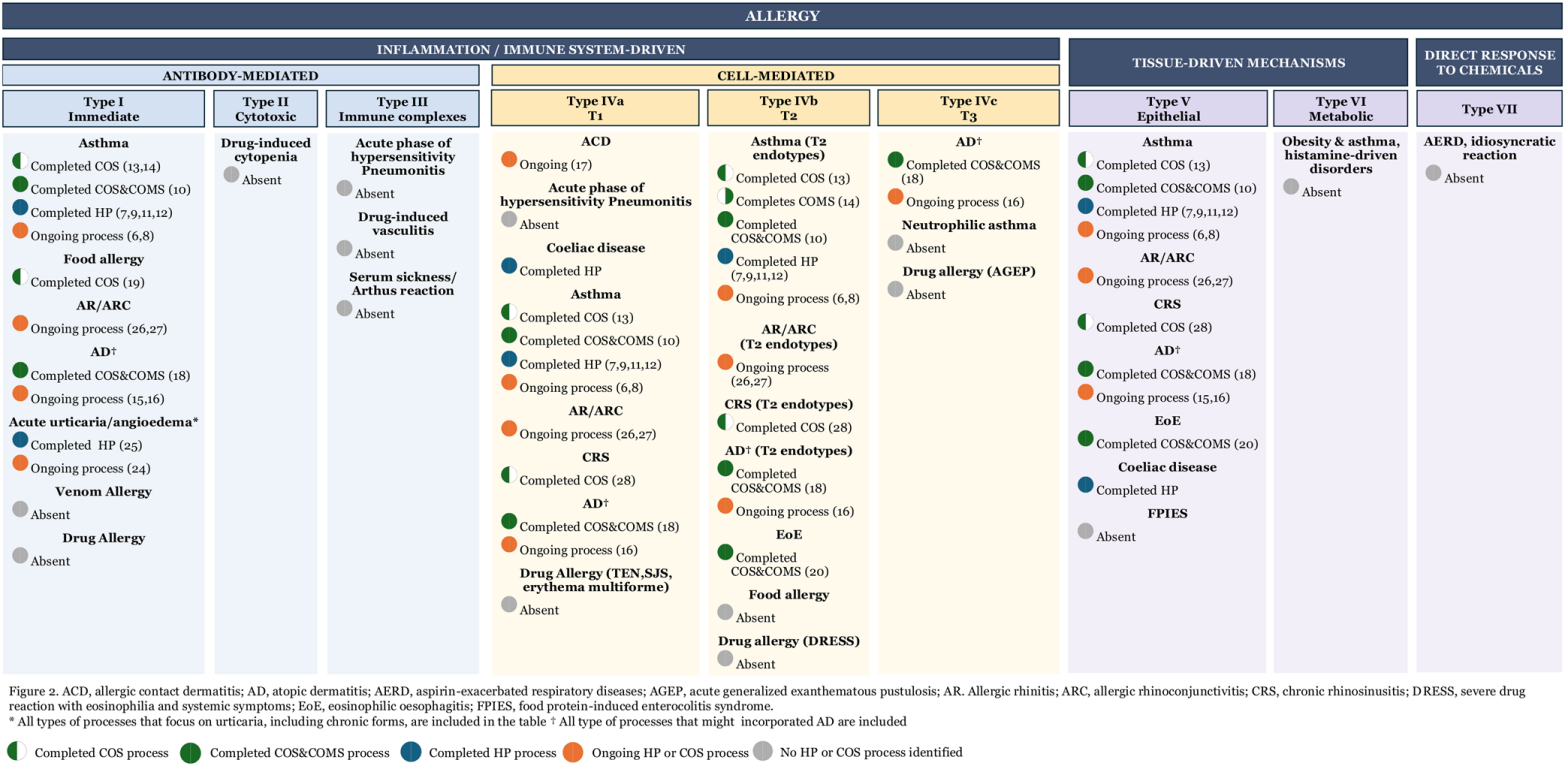
Availability of core outcome sets and outcome harmonisation processes in allergy and clinical immunology mapped over the European Academy of Allergy and Clinical Immunology (EAACI) classification.

The availability of COS and HP varied substantially across the field (Figure 2). Asthma was by far the condition most frequently targeted for the harmonisation efforts, with seven projects completed [7, 9, 10, 11, 12, 13, 14] and two [6, 8] currently under development. In contrast, only one process for hereditary angioedema was identified for clinical immunology [25] and none for anaphylaxis and drug allergy. Other initiatives, developed or currently under development, included eczema [15, 16, 17, 18] urticaria [26, 27], allergic rhinitis [28, 29], chronic rhinosinusitis [30], celiac disease [31], immunoglobulin E (IgE)‐mediated food allergy [19] and eosinophilic esophagitis [20].

Twelve out of 14 initiatives targeted both adults and children. One process focused exclusively on the paediatric population [13], and the other exclusively on adults [30]. COS were focused on: clinical research [7, 10, 13, 14, 15, 19, 20, 25, 27], dual research‐clinical practice settings [12, 18, 27, 32, 33, 34], or clinical practice alone [9, 11]. Detailed information on recommended outcomes/OMI for each included process is available in Table 1.

**TABLE 1:** Core outcome sets and outcome harmonisation processes in allergy and clinical immunology.

Population	Domain/outcome	Measurement instruments		Participants involved
**Asthma**				
**Sinha et al**. [13]				
**Development of a core outcome set for clinical trials in childhood asthma: a survey of clinicians, parents, and young people**				
**COS process** (*pharmacological intervention in clinical trials*)				
Children	Symptoms	COMS process has not been developed yet		Patient perspectives Healthcare professionals/researchers
	Exacerbations			
	Quality of life			
	Mortality			
**Tejwani V et al**. [14]				
**A multistakeholder Delphi consensus core outcome set for clinical trials in moderate‐to‐severe asthma (coreASTHMA)**				
**COS process** (*pharmacological intervention in moderate‐to‐severe asthma in clinical trials*)				
Adults, children	Severe asthma exacerbation	Events requiring systemic corticosteroids^b^ for ≥ 3 day and/or a hospitalisation/emergency room visit for asthma requiring systemic corticosteroids^e^		Patient representatives Healthcare professionals/researchers. Other: regulators, health‐technology assessor, industry
	Change in asthma control	Change in the extent to which the various manifestations of asthma are reduced or removed by treatment. Several tools have been proposed (e.g., ACT, ACQ, and ATAQ) as examples of available tools to measure and describe asthma control^e^		
	Asthma‐specific (or severe asthma‐specific) QoL	Several questionnaires have been proposed by authors, such as ABP, AQLQ‐S, Mini‐AQLQ, LWAQ, Modified AQLQ‐Marks, SGRQ, SAQ#		
	Asthma‐specific hospital stay or admission	Being in a hospital bed, in any setting (inpatient, ICU, emergency), for 24 h or longer, or being admitted regardless of the duration owing to asthma#		
	Asthma‐specific emergency department visit	The number of emergency department visits owing to asthma#		
**Khaleva et al**. [10]				
**Development of Core Outcome Measures sets for paediatric and adult Severe Asthma (COMSA)**				
**COMS process** (*severe asthma biological therapies in clinical trials*)				
Adults, children	Lung function and related measurements	FEV_1_		Patient representatives Healthcare professionals/researchers Other: regulators, industry
	Asthma‐specific quality of life	SAQ (adults); PAQLQ (children)		
	Asthma control	ACQ‐6 (report as the ACQ −5 to describe symptoms and rescue medication use separately) (adults) ACT (children 12–18 years); C‐ACT (children 4–11 years)		
	Healthcare utilisation	Maintenance of oral corticosteroid use		
		Severe exacerbations		
**Reddel et al**. [12]				
**An Official American Thoracic Society/European Respiratory Society Statement: Asthma Control and Exacerbations Standardising Endpoints for Clinical Asthma Trials and Clinical Practice**				
**HP process** (*clinical trials and clinical practice assessing treatment effect on current clinical control*)				
Adults, Children ≥ 6 y.o.	Symptom‐free days	Specific tools not defined explicitly		Working group members (without involvement of patient representatives)
	Reliever use			
	Composite scores			
	Exacerbation (*within last 1–4 wks*.)	Severe exacerbations—events requiring systemic corticosteroids for ≥ 3 day and/or a hospitalisation/emergency room visit for asthma requiring systemic corticosteroids		
	Quality of life	Specific tools not defined		
**HP process** (*clinical trials and clinical practice assessing treatment effect on future risk*)				
Adults, Children ≥ 6 y.o.	Exacerbations	Same definition as above in this process		Working group members (without involvement of patient representatives)
	Post‐BD FEV1 (*for assessment of lung function decline*)	FEV1 recorded 15 min after administration of 400 μg of albuterol or equivalent		
	Composite scores	Specific tools not defined		
	Treatment side‐effects	Record side effects relevant to study medication(s), as‐needed medications, or exacerbation medications, and any withdrawals due to adverse events		
	Pre‐BD FEV1 (*as predictor for exacerbations*)	FEV1 recorded after appropriate withholding of SABA and LABA, if used		
**Busse et al**. [7]				
**Asthma outcomes workshop: Overview**				
**HP process** (*clinical research*)				
**Adults, children**	Exacerbations	Systemic corticosteroids for asthma; Asthma‐specific hospital admissions; Asthma‐specific ED visits; Asthma‐specific ICU admissions/intubations^c^; Death (all cause and asthma related)^c^		Working group members (without involvement of patient representatives)
	Healthcare utilisation and costs	Asthma‐specific hospital admissions; Asthma‐specific ED visits; Asthma‐specific outpatient visits; Asthma‐specific detailed medication use (name, dose, and duration); Resource use related to the intervention (e.g., personnel time, mite eradication, and equipment)		
	Biomarkers	Specific tools not defined		
	Pulmonary physiology	Spirometry		
	Composite scores of asthma control§	ACQ or ACT (adults and adolescents ≥ 12 y.o.); cACT (children 5–11 y.o.)		
	Symptoms	Specific tools not defined		
	Quality of life	Specific tools not defined		
Gliklich et al. [9]				
**Harmonised outcome measures for use in asthma patient registries and clinical practice**				
**HP process** (*patient registries and clinical practice*)				
Adults, Children	Survival	Death (asthma‐related)	Death from asthma reported in 12‐month intervals	Working group members (without involvement of patient representatives)
	Clinical response	Exacerbation	An exacerbation includes prescribed systemic steroids (defined as > 2 day of oral steroids or a steroid injection) or an increase in the oral steroid dose from the baseline dose; or an asthma‐related hospitalisation/ED/urgent care centre unscheduled office visit, or visit requiring a prescription of systemic corticosteroids; or provider documentation of an acute asthma exacerbation	
		Change in asthma control	ACT or ACQ or ATAQ (adults); mTRACK or C‐ACT or ATAQ or ACQ (children)	
		Prebronchodilator indices	Prebronchodilator FEV1 and FVC percent predicted and FEV1/FVC ratio change in measurements over 12‐month period	
		Change in asthma controller medication use	Measured by patient/caregiver self‐report, physician report, prescription fill, or electronic medication monitoring	
		Change in quick‐relief asthma medication use	Measured by patient/caregiver self‐report, physician report, prescription fill, or electronic monitoring	
	Events of interest	Systematic corticosteroids for asthma	Prescriptions for systemic steroids filled within 7 days of a health care visit for asthma and counted as number of events per patient in a 12‐month reporting period	
		Asthma‐specific ED visits	Number of ED visits per patient in the 12‐month reporting period	
		Asthma‐specific hospital admission	Number of hospital admissions caused by asthma per patient in the 12‐month reporting period	
		Near‐fatal asthma	Asthma exacerbation associated with severe respiratory compromise requiring intubation or non‐invasive positive‐pressure ventilation (to prevent it from progressing to a fatal asthma exacerbation)	
		Medication‐related adverse events	Adverse events related to asthma medications	
	Patient reported	Asthma control	Examples of scales provided by authors (PROMIS Global 10 and VR‐12)	
		Medication adherence	Measured by patient/caregiver self‐report, physician report, prescription fill, or electronic medication monitoring	
		Asthma‐specific quality of life	Specific tools not defined	
		General quality of life	Specific tools not defined	
	Resource utilisation	Missed school days/missed workdays	Missed school days: > 1 missed school days caused by asthma in the past 12 m. or number of days missed from school (preferably days missed because of asthma) Missed workdays: caregiver has had > 1 missed day. of work caused by their child's asthma in the past 12 m. or patient has missed > 1 day of work because of asthma—WPAI to count work absence days	
		Asthma medication ratio	The number of asthma controller canisters dispensed in the year divided by the total asthma medication canisters (controllers + relievers) dispensed in the year	
		Unscheduled visits to primary care physician's office/visits to urgent care centre/ED visits/hospital admission for asthma	In the 12‐month reporting period	
		Treatment‐related resource utilisation	All resource utilisation (as measured by cost) related to treatment or management of asthma, including hospitalisations, ED/urgent care centre/office visits, medications, and other costs	
	Experience of care	Patient satisfaction with care	Specific tools not defined	
Martínez‐Moragón et al. [11]				
**Patient‐reported outcome measures in severe asthma: an expert consensus** ^d^				
**HP process** (*severe asthma in clinical practice*)				
Adults, Children	Not explicitly specified^d^	ACT	Patient representatives Healthcare professionals/researchers	
		TAI		
		mini‐AQLQ		
		mMRC		
		Dispensing register		
		EQ‐5D		
		MMAS		
**Atopic eczema**				
**Williams et al**. [18]				
**The HOME Core outcome set for clinical trials of atopic dermatitis**				
**COS process** (*clinical trials*)				
Adults, children	Clinician‐reported signs	EASI	Patient representatives Healthcare professionals/researchers Other: industry representatives, methodologists	
	Patient‐reported symptoms	POEM and NRS11 for itch intensity		
	Quality of Life	DLQI (adults); CDLQI (children); IDQoL (infants)		
	Long‐term control	RECAP or ADCT		
**Jacobson et al. and Leshem et al**. [32, 33, 34]				
**List of feasible validated instruments** (*clinical care*)				
Adults, children	Clinician‐reported signs	IGA multiplied or measured alongside BSA or EASI or vIGA‐AD	Patient representatives Healthcare professionals/researchers Other: industry representatives, methodologists	
	Patient‐reported symptoms	PO‐SCORAD, POEM, Peak 24‐h NRS‐itch and Average 1‐week NRS‐itch (PROMIS Itch Questionnaire)		
	Long‐term control	RECAP or ADCT		
	Eczema‐specific quality of life	Work ongoing		
	Patient global assessment	Work ongoing		
**Food allergy**				
**Demidova et al**. [19]				
**Core Outcome Set for IgE‐mediated food allergy clinical trials and observational studies of interventions: International Delphi consensus study ‘COMFA’**				
**COS process** (*clinical trials and observational studies*)				
Adults, Children	Quality of life	COMS process has not been developed yet	Patient representatives Healthcare professionals/researchers Other: industry representatives	
	Allergic symptoms			
**Ma et al**. [20]				
**Development of a core outcome set for therapeutic studies in eosinophilic esophagitis (COREOS)**				
**COS process** (*clinical trials and observational studies of pharmacologic and diet interventions*)				
Adults, Children	Histopathology	Peak oesophageal eosinophilia and histologic remission	Patient representatives Healthcare professionals/researchers	
	Endoscopy	EREFS		
	Patient‐reported symptoms^a^	DSQ and EEsAI (adults); PEESS v2.0 (children)		
	Quality of life^a^	EoE QoL‐A (adults); PedsQL EoE module (children)		
**Coeliac disease**				
**Ludvigsson et al**. [31]				
**Outcome measures in coeliac disease trials: the Tampere recommendations**				
**HP process** (*clinical trials*)				
Adults, Children	Histology	Histological evaluation should follow a priori histology protocols using quantitative measures	Working group members (including patient representatives)	
	Serology	IgA TG2 and IgG DGP		
	Clinical outcome assessment (including patient‐reported outcomes)	Specific tools not defined		
	Health‐related Quality of life	Specific tools not defined		
**Urticaria**				
**Baiardini et al**. [27]				
**Recommendations for assessing patient‐reported outcomes and health‐related quality of life in patients with urticaria: a GA2LEN taskforce position paper**				
**HP process** (*clinical trials and clinical practice*)				
Adults, Children	Symptoms	UAS	Working group members (without involvement of patient representatives)	
	Quality of life	CU‐Q2oL		
Chronic rhinosinusitis				
**Hopkins et al**. [30]				
**CHronic Rhinosinusitis Outcome MEasures (CHROME) developing a core outcome set for trials of interventions in chronic rhinosinusitis**#				
**COS process** (*clinical trials*)				
Adults	Patient‐reported symptoms and QoL	Overall symptom severity	SNOT‐22 repeated over time**#**	Patient representatives Healthcare professionals/researchers
		Frequency of symptoms	Additional question required to address frequency of symptoms#	
		Duration of symptoms	Specific tools not defined	
		Duration of treatment effect	Specific tools not defined	
		Sense of smell	Specific tools not defined	
		Runny nose/Nasal discharge (anterior or posterior)	Specific tools not defined	
		Nasal obstruction/blockage/congestion	Specific tools not defined	
		Disease‐specific QoL	Specific tools not defined	
	Control of disease	Overall control of disease	Need for systemic medication (steroid or antibiotic)^e^	
		Need for surgery	Progression to surgery^e^	
		Endoscopic appearances (including presence/quality of pus, presence and size of polyps, oedema, crusting, inflammation)	Lund‐Kennedy score^e^	
	Impact on daily activity	Ability to perform normal activities	SNOT‐22 (or specific measures of productivity)^e^	
	Acceptability of treatment and side effects	Compliance with treatment	Measurement of compliance and side effects^e^	
		Acceptability of treatment		
		Side effects of the treatment (including medical and surgical)		
Hereditary angioedema				
**Petersen et al**. [25]				
**A core outcome set for efficacy of acute treatment of hereditary angioedema**				
**COS process** (*acute treatment of attacks in clinical trials*)				
Adults, Children	Change in overall symptom severity at one predetermined point between 15 min and 4 h after treatment	COMS process has not been developed yet	Patient representatives Healthcare professionals/researchers Other: regulators, industry representatives	
	Time to end of the progression of all symptoms			
	Need for rescue medication during the entire attack			
	Impairment of daily activities			
	Treatment satisfaction			

^a^ No instruments for these outcomes met consensus thresholds for use in all observational studies.

^b^ Use of oral systemic corticosteroids or an increase from a stable maintenance dose, for at least 3 days. For consistency, courses of corticosteroids separated by 1 week or more should be treated as separate severe exacerbations. A corticosteroid injection is also considered a discrete use of systemic corticosteroids.

^c^ Should be reported in clinical trials only § no outcome measure was recommended for use in clinical trials for children (5–11 y.o.).

^d^ The process was focused on achieving consensus on the most relevant patient‐reported measurement set, rather than outcomes.

^e^ The COMS was not developed by the authors. The table presents recommendations provided by the authors, rather than the COMS recommendations established through the consensus process.

Studies employed varied methodologies for outcome selection, including Delphi surveys and/or consensus meetings, and stakeholder consultations. The Supporting Information provides detailed methodology of the included studies (Tables S5, S6).

Across the 14 completed initiatives, COS/COMS demonstrated higher adherence to COS‐STAD criteria than HP (median 12/12 criteria met [range 9–12] vs. median 6/12 [range 3–9]; Table S3). Lower adherence among HP was most commonly driven by limited patient involvement and the absence of pre‐specified scoring/consensus procedures.

### 3.3 Asthma

Nine initiatives (five COS [6, 8, 10, 13, 14] and four HP [7, 9, 11, 12]) were designed to address asthma (Table 1). All three finalised COS were developed for clinical trials, each targeting specific subpopulations. The COS for moderate‐to‐severe asthma (coreASTHMA) [14] and the COMS for severe asthma (COMSA) [10] were both designed for use in adult and paediatric populations. However, the COS developed by Sinha et al. [13] was specifically intended for clinical trials in paediatric asthma only.

The earliest COS initiative was conducted by Sinha et al. [13] targeting all childhood asthma clinical trials; four measurement outcomes were suggested: ‘symptoms’, ‘exacerbations’, ‘quality of life’ (QoL), and ‘mortality’, but no COMS process has ever been completed. CoreASTHMA [14] was completed in 2021 and agreed that the following outcomes should be used in clinical trials of pharmacological interventions for moderate‐to‐severe asthma: ‘severe’ asthma exacerbation defined by events requiring systemic corticosteroids for ≥ 3 days and/or hospitalisation/emergency room visit for asthma requiring systemic corticosteroids, ‘asthma‐specific hospital stay or admission’ assessed as a stay in a hospital bed, in any setting for ≥ 24 h, or being admitted regardless of the duration owing to asthma, and the number of ‘asthma‐specific emergency department visits’, ‘change in asthma control’, and ‘asthma‐specific (or severe asthma‐specific) QoL’. While coreASTHMA provides examples of existing instruments that can be used to measure ‘change’ in asthma control and ‘asthma’‐specific (or severe asthma‐specific) ‘QoL’, it was highlighted that current measures of asthma‐specific QoL are not considered to accurately capture the patient experience and further work is needed to address this issue.

COMSA [10] aimed specifically at severe asthma biological therapies and acknowledged building upon the coreASTHMA project, in particular to focus on closing the gaps for the outcomes where the measurement instruments have not been agreed upon. The recommended COS included ‘lung function’ measured with forced expiratory volume in one second (FEV1), ‘asthma‐specific QoL’ assessed by Severe Asthma Questionnaire (SAQ) in adults and Paediatric Asthma Quality of Life Questionnaire (PAQLQ) for children, ‘asthma control’ investigated by Asthma Control Questionnaire (ACQ‐6) for adults (should be reported as the Asthma Control Questionnaire‐5 to describe symptoms and rescue medication use separately), Asthma Control Test (ACT) for children aged 12–18 years, and Childhood Asthma Control Questionnaire (C‐ACT) for the paediatric population aged 4–11 years, as well as ‘healthcare utilisation measures’ estimated via maintenance oral corticosteroid use and severe exacerbations.

Two other COS processes are ongoing: one with Paediatric Emergency Research Networks (PERN) [8], which aims at clinical trials of interventions for acute severe paediatric asthma, and another that looks into TCM interventions [6].

All three finalised processes included multiple rounds of Delphi, while coreASTHMA and COMSA also held consensus meeting(s) with substantial involvement from patient representatives throughout. Industry was involved in the voting process (< 10% of voting participants) in coreASTHMA and COMSA, but not Sinha et al. [13]. Both coreASTHMA and COMSA met all COS‐STAD criteria (Appendix S1, Table S3), while Sinha et al. [13] COS met nine out of eleven. COMSA was the only process in asthma appraising measurement instruments using COnsensus‐based Standards for the selection of health Measurement Instruments (COSMIN) methodology.

Apart from COS initiatives, four HP were developed across this time period. A diverse range of asthma outcomes and OMI were proposed across them. While some outcomes, such as ‘exacerbations’, ‘asthma control’, ‘QoL’, and ‘healthcare utilisation’, were consistently highlighted across different frameworks, others, like biomarkers, medication adherence, and patient satisfaction, were included in select processes. Exacerbations were defined as requiring systemic corticosteroids for at least 3 days and/or hospitalisation or emergency room visits (19), while asthma control was assessed using composite scores (8, 20), changes in medication use, and pulmonary function tests (FEV1/FVC ratios). Suggestions regarding the ‘healthcare utilisation’ assessment included emergency department visits, hospital admissions, unscheduled physician visits, and treatment‐related resource use, whereas no QoL instrument is recommended as a core. One process [9] also incorporated broader patient‐centred outcomes, such as missing school or workdays and patient satisfaction with care. One included HP focused on achieving consensus on the most relevant patient‐reported measurement set [11], rather than outcomes. The recommendations included using the following measures (ACT, TAI, mini‐AQLQ, mMRC, dispensing register, EQ‐5D, MMAS) in clinical practice in severe asthma.

### 3.4 Eczema

Four initiatives focus on eczema, with two addressing atopic dermatitis/eczema and two focusing on hand eczema and eczema more broadly. The Harmonising Outcome Measures for Eczema (HOME) initiative previously developed a COS for clinical trials [18] and is developing a ‘pick and choose’ list of feasible instruments, covering adult and paediatric clinical practice settings. Another ongoing initiative is developing a COS for Traditional Chinese Medicine (TCM) interventions for atopic dermatitis [16].

The other two initiatives focused on hand eczema and eczema in general are ongoing with one developing a COS for hand eczema [17], while the other is focused on TCM interventions for eczema [15].

The HOME initiative [18] included three initial rounds of Delphi surveys, followed by 10 meetings, with strong engagement from patient representatives, with 13% participating in Delphi surveys and an average of 15% in subsequent consensus meetings, respectively. HOME firmly adhered to all COS‐STAD criteria. All outcome measurement instruments were appraised using COSMIN methodology. HOME COS for clinical trials included ‘clinician‐reported signs’ measured by the Eczema Area and Severity Index (EASI), ‘patient‐reported symptoms’ assessed through the Patient‐Oriented Eczema Measure (POEM), peak 11‐point numerical rating scale (NRS‐11) in the past 24 h for ‘itch intensity’ and ‘long‐term control’ measured by Recap of atopic eczema (RECAP) or Atopic Dermatitis Control Tool (ADCT). Recommended measures for evaluating ‘QoL’ were the Dermatology Life Quality Index (DLQI) for adults, the Children's Dermatology Life Quality Index (CDLQI) for children, and the Infants' Dermatitis Quality of Life Index (IDQoL) for infants.

For clinical care, HOME developed a list of feasible, validated instruments from which clinicians can pick and choose, depending on their needs and preferences. These included ‘clinician‐reported signs’ measured by Investigator's Global Assessment (IGA) multiplied by or measured concurrently with a body surface area (BSA), EASI, or Validated Investigator Global Assessment for Atopic Dermatitis (vIGA‐AD); patient‐reported symptoms assessed by Patient‐Oriented SCORing for Atopic Dermatitis (PO‐SCORAD), POEM, Peak 24‐h Peak Pruritus Numerical Rating Scale(NRS‐itch), and Average 1‐week NRS‐itch using the Patient‐Reported Outcomes Measurement Information System (PROMIS) Itch Questionnaire; and disease control measured by RECAP or ADCT. A workaround for identifying appropriate measurements for eczema‐specific QoL and patient global assessment in clinical practice is ongoing.

### 3.5 Food Allergy

Two COS development processes are conducted in relation to food allergy. The Core Outcome Measures for Food Allergy (COMFA) [19] covered IgE‐mediated food allergy, while the core outcome set for therapeutic studies in eosinophilic esophagitis (COREOS) [20] is the only project covering any type of non‐IgE‐mediated food allergy.

COMFA [19] developed a COS for clinical trials and observational studies and reached an agreement that ‘allergic symptoms’ and ‘QoL’ should be measured. COMFA followed all COS‐STAD criteria and methodology included a systematic review of previously used outcomes, pre‐Delphi workshops, a two‐stage Delphi process, and a consensus meeting. Patient representatives participated throughout the process, with 53% representation in the Delphi process and 20% in a consensus meeting. Industry was not involved in the decision‐making process. The COMS process has not been launched, and the OMI for core outcomes is yet to be defined.

COREOS [20] aimed at clinical trials and observational studies of pharmacological and dietary interventions and recommended measuring ‘histopathology’ with peak oesophageal eosinophilia and histologic remission, ‘endoscopy’ with Eosinophilic Esophagitis Endoscopic Reference Score (EREFS), ‘patient reported symptoms’ with Dysphagia Symptoms Questionnaire (DSQ) and Eosinophilic Esophagitis Activity Index (EEsAI) for adults and Paediatric Eosinophilic Esophagitis Symptom Score (PEESS v2.0) for children, and ‘QoL’ with eosinophilic QoL questionnaire (EoE‐QoL‐A) in adults and PedsQL EoE Module for children. To inform the long list of outcomes, literature systematic reviews were conducted as well as surveys of people with lived experience among 190 individuals. The outcome voting process consisted of an expert Delphi and consensus meeting (however, patient representatives were not involved). COREOS met all COS‐STAD criteria but one (it was unclear whether ambiguity of language used in the list of outcomes was avoided). Industry was not involved in the voting process.

### 3.6 Coeliac Disease

One HP [31] was conducted for coeliac disease aimed to standardise outcomes in clinical trials for both paediatric and adult populations. Recommended outcomes include ‘histology’, ‘Serology with IgA TG2 and IgG DGP assessment, ‘clinical outcome assessment (including patient‐reported outcomes)’, and ‘health‐related QoL’.

The methodology included a systematic literature review and a task force group that provided recommendations with two patient representatives among the experts. Industry did not participate in the process, and seven out of eleven COS‐STAD criteria were met.

### 3.7 Urticaria

The Global Allergy and Asthma European Network (GA^2^LEN [27] conducted a single HP for urticaria), aimed at clinical trials and clinical practice, covering all age groups. Recommended outcomes that need to be assessed include ‘symptoms’ measured with Urticaria Activity Score (UAS) and ‘QoL’ assessed by the Chronic Urticaria QoL Questionnaire (CU‐Q2oL). The methodology included expert discussions with no involvement from patient representatives. Industry did not participate in the process, and four out of eleven COS‐STAD criteria were met.

### 3.8 Allergic Rhinitis

We found two COS initiatives aimed at allergic rhinitis. Both were ongoing and focused on developing COS for TCM interventions [28, 29].

### 3.9 Chronic Rhinosinusitis

The Chronic Rhinosinusitis Outcome MEasures (CHROME) initiative [30] developed a COS for chronic rhinosinusitis. The recommended core set consisted of 15 outcomes over four domains. While a COMS has not been conducted by the initiative, some provisional recommendations regarding how to measure these outcomes were given by the steering committee.

The CHROME initiative [30] included a systematic literature review, a patient and practitioners' survey to inform the long list, and a two‐round Delphi survey. Patients' representatives were involved throughout the process, from identifying the potential outcomes to reviewing definitions and participating in the Delphi process. CHROME met all COS‐STAD criteria.

### 3.10 Other Allergic Diseases

As for other allergic conditions, we failed to identify COS or HP for venom allergy, drug allergy, induced drug‐cytopenia, acute phase of hypersensitivity pneumonitis, drug‐induced vasculitis, serum sickness, and FPIES.

### 3.11 Clinical Immunology

Across clinical immunology, only one COS has been developed. The Acute Treatment Outcomes in Hereditary Angioedema (AURORA) project [25] developed a COS aimed at clinical trials. ‘Change in overall symptom severity at one predetermined point between 15 min and 4 h after treatment’, ‘time to end of the progression of all symptoms’, ‘need for rescue medication during the entire attack’, ‘impairment of daily activities’, and ‘treatment satisfaction’ were the recommended outcomes. OMI for these outcomes has not yet been defined. The methodology included a systematic review and a Delphi process involving patient representatives. Industry participated in the voting and decision‐making process. AURORA met nine out of eleven COS‐STAD criteria.

## 4 Discussion

The findings of this systematic review highlight both the progress made and the ongoing challenges in the COS development and HP in allergy and clinical immunology. Despite a growing number of initiatives aimed at outcome reporting standardisation, substantial variability remains in the methodology employed and in the degree of adherence to established COS development standards. While conditions such as asthma and atopic eczema have received considerable attention, significant gaps remain regarding other allergic and immunological conditions, including drug allergy and anaphylaxis. Although many COS initiatives incorporate patient perspectives and stakeholder consensus, actual incorporation of such viewpoints [35] in clinical research and practice is inconsistent, thus limiting their impact. These findings highlight the need for harmonisation efforts that ensure methodological consistency, wider disease coverage, and stronger patient involvement, alongside greater engagement with regulatory bodies and policymakers to support COS adoption in research and practice.

**FIGURE 3:**
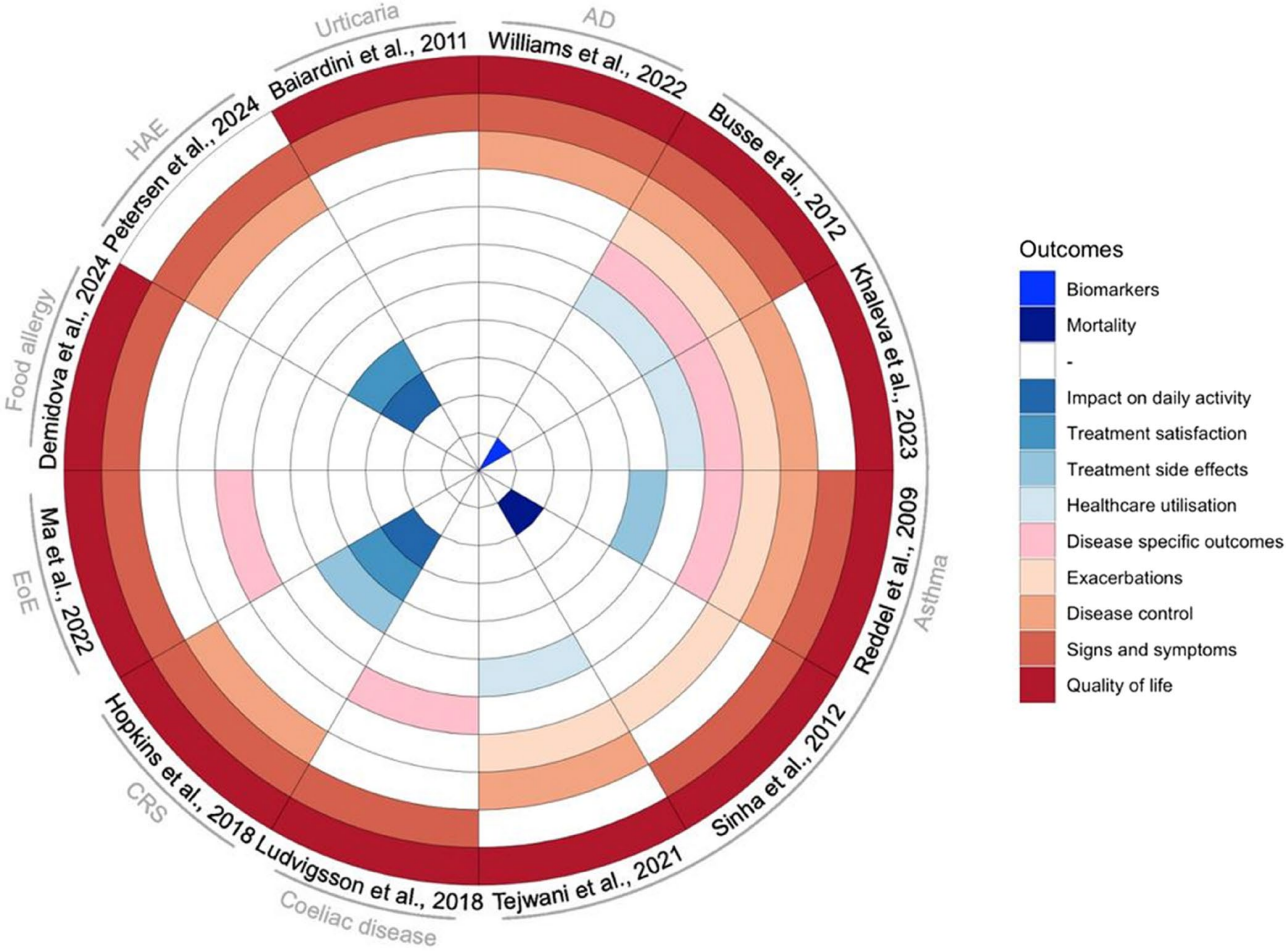
‘Core’ outcomes included in the Core Outcome Sets and HP of different allergic and immunologic conditions. Distribution of outcome domains in COS and harmonisation processes for clinical trials across allergic and immunologic conditions. The radial chart shows 12 colour‐coded outcome domains reported across COS and harmonisation initiatives. Domain definitions vary: in Khaleva et al. [10], Healthcare utilisation includes maintenance of oral corticosteroids and severe exacerbations, which are displayed separately here. In Tejwani et al. [14] and Busse et al. [7], this domain refers to asthma‐specific hospital and emergency visits. In Petersen et al. [25], outcomes for acute attacks were separated into Symptoms and Disease control. Details of outcome grouping are available in Appendix S1, Table S7.

Some key themes emerged across COS developed in allergy and clinical immunology, reflecting shared priorities and preferences across stakeholders regardless of the disease they represent. ‘QoL’ and ‘symptoms’ were consistently prioritised, highlighting the importance of patient‐centred outcomes that address both subjective experiences and clinical manifestations (Figure 3). Beyond their clinical relevance, measures of quality of life (QoL) are also important in calculating quality‐adjusted life years (QALYs), a key metric in cost‐effectiveness analyses of clinical interventions [36]. ‘Exacerbations’ and ‘disease control’ were also common among critically important outcomes, particularly in asthma‐related COS, reflecting their relevance in monitoring chronic disease progression and treatment efficacy. Regulatory agencies, such as the European Medicines Agency and the U.S. Food and Drug Administration, have historically emphasised investigator‐reported outcomes, considering them more objective and reliable than patient‐reported outcomes [37]. In fact, the FDA does not accept patient‐reported outcomes as a stand‐alone primary measure of efficacy, whereas the EMA does. This preference arises from concerns about the potential subjectivity and variability inherent in patient‐reported outcomes, which can introduce data interpretation and consistency challenges. However, there is an obvious growing body of evidence [38] of the value that patient‐reported outcomes bring to understanding the patient's perspective, as seen in recent guidance from regulatory agencies [39] encouraging the use of validated patient‐reported outcome measures.

Several COS and HP were developed for asthma clinical trials, each targeting different patient populations and treatment approaches. While they varied in scope and recommended outcome measurement instruments, all consistently prioritised ‘exacerbations’ and ‘QoL’ as core outcomes. This may reflect the clinical and economic importance of exacerbations, which are readily measurable and frequently used in trials and guidelines, and the wide availability of validated QoL tools, which have become standard for capturing patient‐reported outcomes. However, despite alignment on these key outcomes, it remains unclear to what extent COS has been adopted into clinical trials and thus translated into clinical guidelines, which may limit their broader implementation in research and care. Although each COS and some HP aimed at a slightly different aspect, the number of initiatives is somehow worrying as outcome harmonisation initiatives are aiming at standardisation. However, availability of multiple COS/HPs may bring even more heterogeneity, with trialists lost between the guideline options being unsure of which one is best to implement [40]. Given the risk that multiple overlapping initiatives may generate further heterogeneity, coordinated prioritisation is needed. We suggest that EAACI and/or GA^2^LEN, in collaboration with COMET, could facilitate a coordination forum to (i) map existing sets, (ii) agree on which COS/COMS should be considered ‘reference’ for key trial contexts, and (iii) schedule planned updates aligned with therapeutic advances.

Beyond development quality, uptake remains a key challenge. Barriers include limited awareness among trialists, perceived burden of additional data collection, uncertainty when multiple overlapping recommendations exist, and, critically, the absence of agreed measurement instruments for some core outcomes. Practical strategies to increase utilisation include embedding COS/COMS in trial registry templates and funding calls, journal endorsement and enforcement (e.g., requiring reporting against COS where available), and aligning COS/COMS outputs with registries and guideline evidence‐to‐decision frameworks.

The harmonisation of core outcomes in eczema, urticaria, and hereditary angioedema has made significant progress, yet challenges in standardisation, implementation, and regulatory alignment persist [35]. The HOME [18] initiative is one of the most well‐developed and exemplary initiatives across medical fields. It has successfully established core outcomes for eczema clinical trials and a list of feasible, validated instruments, incorporating clinician‐reported signs, patient‐reported symptoms, long‐term control and QoL measures.

While COS frameworks are well‐established for asthma and atopic eczema, they are still lacking for conditions such as drug allergy, anaphylaxis, venom allergy, and drug‐induced hypersensitivity reactions, which is surprising considering their high prevalence and health economic impact. Possible reasons include phenotypic heterogeneity, variable triggers and care pathways, and the episodic/acute nature of events such as anaphylaxis, which reduces interventional trial volume and complicates standardised follow‐up windows. These areas also span multiple research communities (allergy, emergency medicine, dermatology, pharmacovigilance), which can fragment consensus‐building. In addition, existing standardisation efforts have often focused on case definitions and severity grading rather than consensus‐driven core outcome domains and measurement instruments. The absence of COS for drug‐induced cytopenia, acute hypersensitivity pneumonitis, drug‐induced vasculitis, serum sickness, and FPIES suggests a potential assessment of the need for further research efforts in these areas. For eosinophilic esophagitis, the COREOS [20] initiative reached an agreement on several critical outcomes, but the COMFA [19] initiative reached a consensus regarding outcome domains; yet measurement instruments for these outcomes remain undefined, limiting clinical trial implementation and observational research comparability.

Across all the processes suggesting measurement instruments, only a limited number of initiatives [18] evaluated considered instruments using recommended COSMIN methodology [10, 41], ensuring that recommended instruments are valid and reliable. This is not unique to the field as implementation of COSMIN methodology remains a challenge across COS development processes, due to its methodological complexity [42]. COSMIN systematic reviews of outcome measurement properties [43] take very long time to complete, and some COS processes do not incorporate them as a part of the process which raises concerns regarding appropriateness of agreed‐upon instruments use for the outcome assessment. One notable gap is the lack of recall periods associated with outcome measurement instruments. Despite their critical role in ensuring consistency and interpretability of data—particularly for symptoms and quality‐of‐life measures—most initiatives do not specify the time frame over which outcomes should be assessed (e.g., past 24 h, past week). This limits comparability across studies and may undermine the validity of meta‐analyses and guideline recommendations.

Collaboration between COS/HP developers and regulatory bodies remains challenging. This may originate from the importance of an impartial stance by the regulators, which is a backbone of transparent and unbiased decision‐making. However, the lack of interaction between academics, attempting to harmonise outcomes, and regulators, guiding the process via formal advice on the appropriateness of outcomes and measurement instruments/definitions for their assessment may result in redundant activities and lack of COS implementation. Future COS development should enhance engagement from patient advocacy groups, regulatory agencies, policy makers, healthcare professionals, methodologists, and trialists to ensure that outcome measures are clinically meaningful, patient‐centred, and widely accepted, while considering potential mitigation strategies to prevent conflict of interest influence on the decision‐making process.

Multistakeholder engagement is another area for further improvement (Table S6). Handling of industry involvement in the initiatives differed substantially, with some COS initiatives, such as COMSA and AURORA, involving industry stakeholders throughout the process; some invited industry representatives to participate in discussions, but not voting [19] and others excluded them entirely [7, 11, 12, 13, 20, 25, 30]. The COMET Handbook recommends involving a wide range of stakeholders, including industry representatives, in the development of COS to ensure outcomes are relevant and widely applicable. At the same time, it stresses the importance of managing potential conflicts of interest transparently when industry is involved [4]. Industry participation can take many forms and is seen as particularly valuable, given that COS that do not align with regulatory requirements and industry capabilities are unlikely to be adopted in practice. Industry professionals, often scientists and regulatory experts, can significantly contribute to COS development where regulatory involvement is otherwise difficult. However, maintaining independence and avoiding bias is crucial, and while funding from multiple companies without direct content involvement is possible, there is often a risk of hidden influence that must be carefully managed.

A key strength of this systematic review is its comprehensive scope, covering HP and COS and COMS initiatives across the field. By systematically identifying and synthesising these efforts, the review provides a detailed overview of the current landscape, highlighting the most critical outcomes and providing holistic perspective into the unmet needs. We have evaluated initiatives' adherence to COS‐STAD criteria, offering insights into methodological rigour across different initiatives. Despite these strengths, several limitations must be acknowledged. First, the reliance on published literature may have led to the omission of unpublished or ongoing harmonisation efforts, potentially underestimating the true extent of COS development in the field. Additionally, while efforts were made to evaluate methodological quality, the assessment of adherence to COS‐STAD criteria was based on available reports, and specific aspects of the development process may not have been transparently documented. The heterogeneity of included studies, particularly in terms of methodology and intended applications, also posed challenges in synthesis and comparison. This review did not assess the real‐world implementation or uptake of the identified COS in clinical trials and practice, which remains a crucial aspect of their impact. Given heterogeneous terminology across the field (e.g., ‘endpoint standardisation’, ‘outcome measures’, ‘recommendations’), some misclassification between COS and broader HP is possible. We did not assess real‐world uptake/impact of identified initiatives in trials or practice; this requires different methods (e.g., audits of trial registries and published RCTs). Restricting inclusion to English‐language publications may also have introduced language bias.

This systematic review is the very first attempt to map outcome harmonisation activity across allergy and clinical immunology, identifying major gaps in disease coverage and methodological consistency. Future efforts should prioritise neglected areas (including drug allergy, anaphylaxis and venom allergy), strengthen stakeholder inclusivity, and ensure measurement instrument selection is addressed using robust methods. By providing a field‐wide overview of existing initiatives and unmet needs, this review offers a roadmap for coordinated COS/COMS development and implementation in allergy and clinical immunology.

## Author Contributions

A.D., J.L.P.P., P.R.D.R., C.A., R.J.B., J.G.and D.Mu.: conceptualisation. A.D., D.Mu., N.K., K.P., C.A., R.J.B., J.G.: Methodology. N.K., K.P., E.A., M.E., S.K., A.N., I.M., E.E., S.D., N.D.: Data extraction and analysis. A.D., N.K., K.P., E.A., M.E., S.K., A.Nu., I.M., E.E., S.D., N.D., K.P.D., A.A., N.B., E.B., P.C., J.C., D.K.C., M.M.E., L.F., A.D.G., M.Gi., M.Gh., K.R.J., C.J.J., E.K., R.C.K., Y.A.L., D.P.M., I.Ma., M.J.M., D.Mi., A.Nt., C.Ö., D.P., J.L.P.P., P.R.D.R., A.‐M.M.S., A.F.S., S.S., J.U., W.V., J.G., R.J.B., C.A., D.M.: Manuscript drafting. All authors read and approved the final manuscript.

## Funding

The authors have nothing to report.

## Conflicts of Interest

A.D. reports no financial conflicts of interest but acknowledges coordinating role in The Core Outcome Measures for Food Allergy (COMFA) consortium. N.B., E.B., P.C., J.C., D.K.C., M.M.E., A.D.G., M.Gi., M.Gr., C.J.J., D.P.M., I.M., M.J.M., D.Mi., C.Ö., D.P., P.R.D.R., A.F.S., S.S., J.U., W.V., J.G. were involved in the COMFA initiative. E.K. reports coordinating role in COMSA and involvement in COMFA. Matt Greenhawt is a consultant for Aquestive, is a member of physician/medical advisory boards for DBV Technologies, Takeda, Griffols, Nutricia, Novartis, Aquestive, Allergy Therapeutics, AstraZeneca, ALK‐Abello, Bryn, Genentech, and Prota, is a speaker for ARS and Genentech, is an unpaid member of the scientific advisory council for the National Peanut Board and medical advisory board of the International Food Protein Induced Enterocolitis Syndrome Association, is a member of the Brighton Collaboration Criteria Vaccine Anaphylaxis 2.0 working group, is the senior associate editor for the Annals of Allergy, Asthma, and Immunology, and is a member of the Joint Taskforce on Allergy Practice Parameters. C.A. reports personal fees from Dr. Wolff Group, Sanofi, LEO Pharma, IVDK, Incyte, Pfizer, Effik, and Bionorica, and institutional funding from Dr. Wolff GmbH and Bionorica. He also serves as chair of Harmonising Outcome Measures for Eczema (HOME), the Hand Eczema Core Outcome Set (HECOS), and had a leadership role in COMFA. K.P.D. has a coordinating role in the COMFA consortium, the HOME and HECOS initiative and is part‐time employed by the Information Network of Departments of Dermatology (IVDK). Y.A.L. reports honoraria or fees from AbbVie, Sanofi, Janssen, Pfizer, Eli‐Lilly, and Genentech, an independent research grant from AbbVie, and without personal compensation provided investigator services for Eli Lilly, Pfizer, Sanofi, and AbbVie. She is also involved in the HOME initiative. C.J.J. reports research funding from the National Institute for Health Research, the Food Standards Agency, and Innovate UK, and honoraria from the National Institute for Health Research, Mead Johnson, Nutricia, DBV Technologies, and Allergy UK. J.L.P.P. is Section Head, Allied Health, and Co‐Lead, Research Pillar for the Canadian Society of Allergy and Clinical Immunology and is on the steering committee for Canada's National Food Allergy Action Plan. She reports consulting for Ajinomoto Cambrooke, Novartis, Nutricia, and ALK Abelló. She acknowledges competitive research funding from CAAIF, CIHR, Research Manitoba, Health Sciences Centre Foundation (Manitoba), Children's Hospital Research Institute of Manitoba, University of Manitoba, and the Social Sciences and Humanities Research Council of Canada. R.C.K. reports research funding from the National Institute for Health Research, Aimmune, National Peanut Board, Novartis, and the Food Standards Agency, and honoraria from Nutricia, Viatris, Stallergenes, and DBV Technologies. R.C.K. is also Chair of the British Society for Allergy and Clinical Immunology Psychology Special Interest Group. AMS is on an advisory board for ALK and has received speaker fees from ALK and Thermo Fisher Scientific. R.J.B. reports personal fees from the UK Department of Health and Social Care, the Norwegian Directorate of Public Health, the World Health Organization, Wiley publisher, the British Society for Allergy and Clinical Immunology, and Taus, Cebulash and Landau. D.Mu. reports no financial conflict of interest but acknowledges leading role in the COMFA consortium. Other authors do not report relevant conflicts of interest.

## Supporting information


**Appendix S1:** cea70251‐sup‐0001‐AppendixS1.zip.

## Data Availability

The data that support the findings of this study are available from the corresponding author upon reasonable request.
