# Supplementary material for: ‘What’ and ‘How’ to Measure in Allergy and Clinical Immunology: A Systematic Review of Core Outcome Sets and Outcome Harmonisation Processes
Source: Clin Exp Allergy. 2026 Apr 13;56(5):476–97. doi: 10.1111/cea.70251 (PMC13135876; doi:10.1111/cea.70251)
Supplement: Supplementary file 1 — Appendix S1: cea70251‐sup‐0001‐AppendixS1.zip. [file CEA-56-476-s001.zip › cea70251-sup-0001-TableS1-S7@Supplementary material_COS mapping allergy_31.01.2026.docx]

Table of contents

[Table S1. Systematic review search strategy 2](#_Toc196406275)

[Table S2. Core Outcome Set-STAndards for Development (COS-STAD) checklist. 4](#_Toc196406276)

[Table S3. Core Outcome Set-STAndards for Development (COS-STAD) criteria assessment. 5](#_Toc196406277)

[Table S4 COS and Harmonisation processes currently under development. 6](#_Toc196406278)

[Table S5 Methodology used in included HP and COS processes. 7](#_Toc196406279)

[Table S6 Details of the involved stakeholders in the process 9](#_Toc196406280)

[Table S7. Grouping of individual outcomes into predefined domains across COS and harmonisation initiatives for clinical trials in allergic and immunologic conditions. 13](#_Toc196406281)

[References 15](#_Toc196406282)

# Table S1. Systematic review search strategy

| **Embase 03.06.2024** | **Medline 03.06.2024** | **COMET 26.08.2024** |  |
| --- | --- | --- | --- |
| 1.exp food allergy/  2.exp asthma/  3.exp allergic rhinitis/  4.exp urticaria/  5.exp eczema/  6.exp anaphylaxis/  7."allerg*".ab,ti.  8."food allerg*".ab,ti.  9."hypersensitiv*".ab,ti.  10."asthma*".ab,ti.  11."anaphyla*".ab,ti.  12."atopic dermatiti*".ab,ti.  13."eczema*".ab,ti.  14."urticaria*".ab,ti.  15.hives.ab,ti.  16.allergic rhinitis.ab,ti.  17.hay fever.ab,ti.  18.eosinophilic esophagitis.ab,ti.  19.wheeze.ab,ti.  20.exp wheezing/  21.exp eosinophilic esophagitis/  22.((primary or severe or combined or common variable) adj4 immunodeficienc*).ab,kf,ti.  23.immunologic deficiency syndromes/ or agammaglobulinemia/ or common variable immunodeficiency/ or dysgammaglobulinemia/ or hyper-igm immunodeficiency syndrome/ or hyper-igm immunodeficiency syndrome, type 1/ or iga deficiency/ or igg deficiency/ or severe combined immunodeficiency/ or x-linked combined immunodeficiency diseases/  24.(antibod* adj2 deficienc*).ab,kf,ti.  25.((IgA or IgG) adj2 deficienc*).ab,kf,ti.  26.angioedema.ab,kf,ti.  27.allergic contact dermatitis.ab,kf,ti.  28.allergic contact dermatitis/  29.(FPIES or Food protein-induced enterocolitis syndrome or Dietary protein-induced proctitis or allergic proctocolitis or Eosinophilic gastrointestinal disorders or celiac disease).ab,kf,ti.  30.(venom allergy or drug allergy or drug-induced cytopenia or drug-induced vasculitis or serum sickness or Arthus reaction or Steven Johnson Syndrome or Erythema multiforme or Toxic epidermal necrolysis or DRESS or Aspirin-exacerbated respiratory distress or Idiosyncratic reaction*).ab,kf,ti.  31.insect allergy/  32.food protein induced enterocolitis syndrome/  33.eosinophilic gastrointestinal disorder/  34.celiac disease/  35.drug hypersensitivity/  36.Stevens Johnson syndrome/  37.erythema multiforme/  38.toxic epidermal necrolysis/  39.DRESS syndrome/  40.(hereditary angioedema or hypogammaglobulinemia or hemophagocytic lymphohistiocytosis or chronic granulomatous disease or leukocyte adhesion deficiency or autoimmune lymphoproliferative syndrome or immunodysregulation polyendocrinopathy enteropathy or autoimmune polyendocrinopathy candidiasis).ab,kf,ti.  41.angioneurotic edema/  42.immunoglobulin deficiency/  43.chronic granulomatous disease/  44.autoimmune lymphoproliferative syndrome/  45.1 or 2 or 3 or 4 or 5 or 6 or 7 or 8 or 9 or 10 or 11 or 12 or 13 or 14 or 15 or 16 or 17 or 18 or 19 or 20 or 21 or 22 or 23 or 24 or 25 or 26 or 27 or 28 or 29 or 30 or 31 or 32 or 33 or 34 or 35 or 36 or 37 or 38 or 39 or 40 or 41 or 42 or 43 or 44  46.(Core Outcome Set* or Delphi or Core Outcome Measur* or Delphi Technique or Consensus).ab,kf,ti.  47.consensus/  48.consensus development/ or Delphi study/  49.(outcome harmonisation or outcome harmonization).ab,kf,ti.  50.endpoint determination.ab,kf,ti.  51.46 or 47 or 48 or 49 or 50  52.45 and 51 | 1.exp Food Hypersensitivity/  2.exp Asthma/  3.exp Rhinitis, Allergic/  4.exp Urticaria/  5.exp Eczema/ or exp Dermatitis, Atopic/  6.exp Anaphylaxis/  7."allerg*".ab,ti.  8."food allerg*".ab,ti.  9."hypersensitiv*".ab,ti.  10."asthma*".ab,ti.  11."anaphyla*".ab,ti.  12."atopic dermatiti*".ab,ti.  13."eczema*".ab,ti.  14."urticaria*".ab,ti.  15.hives.ab,ti.  16.allergic rhinitis.ab,ti.  17.hay fever.ab,ti.  18.eosinophilic esophagitis.ab,ti.  19.wheeze.ab,ti.  20.exp Respiratory Sounds/  21.exp Eosinophilic Esophagitis/  22.((primary or severe or combined or common variable) adj4 immunodeficienc*).ab,kf,ti.  23.(antibod* adj2 deficienc*).ab,kf,ti.  24.((IgA or IgG) adj2 deficienc*).ab,kf,ti.  25.Immunologic Deficiency Syndromes/  26.Agammaglobulinemia/  27.Common Variable Immunodeficiency/  28.Dysgammaglobulinemia/  29.Hyper-IgM Immunodeficiency Syndrome/  30.Hyper-IgM Immunodeficiency Syndrome, Type 1/  31.IgA Deficiency/  32.IgG Deficiency/  33.Severe Combined Immunodeficiency/  34.X-Linked Combined Immunodeficiency Diseases/  35.angioedema.ab,kf,ti.  36.allergic contact dermatitis.ab,kf,ti.  37.(FPIES or Food protein-induced enterocolitis syndrome or Dietary protein-induced proctitis or allergic proctocolitis or Eosinophilic gastrointestinal disorders or celiac disease).ab,kf,ti.  38.(venom allergy or drug allergy or drug-induced cytopenia or drug-induced vasculitis or serum sickness or Arthus reaction or Steven Johnson Syndrome or Erythema multiforme or Toxic epidermal necrolysis or DRESS or Aspirin-exacerbated respiratory distress or Idiosyncratic reaction*).ab,kf,ti.  39.Bee Venoms/ or "Insect Bites and Stings"/ or Hymenoptera/  40.Celiac Disease/  41.Drug Hypersensitivity/  42.Stevens-Johnson Syndrome/  43.Erythema Multiforme/  44.Drug Hypersensitivity Syndrome/  45.(hereditary angioedema or hypogammaglobulinemia or hemophagocytic lymphohistiocytosis or chronic granulomatous disease or leukocyte adhesion deficiency or autoimmune lymphoproliferative syndrome or immunodysregulation polyendocrinopathy enteropathy or autoimmune polyendocrinopathy candidiasis).ab,kf,ti.  46.Angioedema/  47.Granulomatous Disease, Chronic/  48.Autoimmune Lymphoproliferative Syndrome/  49.1 or 2 or 3 or 4 or 5 or 6 or 7 or 8 or 9 or 10 or 11 or 12 or 13 or 14 or 15 or 16 or 17 or 18 or 19 or 20 or 21 or 22 or 23 or 24 or 25 or 26 or 27 or 28 or 29 or 30 or 31 or 32 or 33 or 34 or 35 or 36 or 37 or 38 or 39 or 40 or 41 or 42 or 43 or 44 or 45 or 46 or 47 or 48  50.(Core Outcome Set* or Delphi or Core Outcome Measur* or Delphi Technique or Consensus).ab,kf,ti.  51.(outcome harmonisation or outcome harmonization).ab,kf,ti.  52.endpoint determination.ab,kf,ti.  53.Delphi Technique/ or Consensus/  54.Endpoint Determination/  55.50 or 51 or 52 or 53 or 54  56.49 and 55 | Search Options:  Disease Name:  Allergic conjunctivitis;  Allergic disease;  Allergic rhinitis;  Asthma ;  Atopic dermatitis;  Atopic Eczema;  Eczema ;  Eosinophilic esophagitis;  Food allergy;  Hereditary Angioedema;  Respiratory allergy;  Severe asthma;  Published/ Unpublished: Both | |

# Table S2. Core Outcome Set-STAndards for Development (COS-STAD) checklist.

| **COS STAD criteria** | | |
| --- | --- | --- |
| 1 | Scope specification | The research or practice setting(s) in which the COS is to be applied |
| 2 |  | The health condition (s) covered by the COS |
| 3 |  | The population(s) covered by the COS |
| 4 |  | The intervention(s) covered by the COS |
| 5 | Stakeholders involved | Those who will use the COS in research |
| 6 |  | Healthcare professionals with experience of patients with the condition |
| 7 |  | Patients with the condition or their representatives |
| 8 | Consensus process | Initial list of outcomes considered both healthcare professionals' and patients' views |
| 9a |  | A scoring process was described a priori |
| 9b |  | A consensus definition was described a priori |
| 10 |  | Criteria for including/dropping/adding outcomes were described a priori |
| 11 |  | Care was taken to avoid ambiguity of language used in the list of outcomes |

# Table S3. Core Outcome Set-STAndards for Development (COS-STAD) criteria assessment.

| Condition | COS/HP | Initiative | Standart N | | | | | | | | | | | |  |
| --- | --- | --- | --- | --- | --- | --- | --- | --- | --- | --- | --- | --- | --- | --- | --- |
|  |  |  | **Scope** | | | **Stakeholders** | | | **Consensus process** | | | | | | **Total criteria met** |
|  |  |  | **1** | **2** | **3** | **4** | **5** | **6** | **7** | **8** | **9а** | **9b** | **10** | **11** |  |
| Asthma | COS | (Sinha et al., 2012) | V | V | V | V | X | V | V | V | X | X | V | V | 9 |
|  |  | (Tejwani et al., 2021) | V | V | V | V | V | V | V | V | V | V | V | V | 12 |
|  |  | (Khaleva et al., 2023) | V | V | V | V | V | V | V | V | V | V | V | V | 12 |
|  | HP | (Reddel et al., 2009) | V | V | V | X | V | V | X | X | X | X | X | X | 5 |
|  |  | (Busse et al., 2012) | V | V | V | 0 | NA* | 0 | X | X | X | X | X | X | 3 |
|  |  | (Martínez-Moragón et al., 2024) | V | V | 0 | 0 | NA* | V | V | V | V | V | V | V | 9 |
|  |  | (Gliklich et al., 2019) | V | V | V | 0 | V | V | V | X | X | X | X | V | 7 |
| Atopic eczema | COS | (Williams et al., 2022) | V | V | V | V | V | V | V | V | V | V | V | V | 12 |
| Food allergy | COS | (Demidova et al., 2024) | V | V | V | V | V | V | V | V | V | V | V | V | 12 |
|  |  | (Collaborators et al., 2022) | V | V | V | V | V | V | V | V | V | V | V | 0 | 11 |
| Coeliac disease | HP | (Ludvigsson et al., 2018) | V | V | V | V | V | V | V | 0 | X | X | X | X | 7 |
| Urticaria | HP | (Baiardini et al., 2011) | V | V | 0 | 0 | V | V | X | X | X | X | X | X | 4 |
| Chronic Rhinosinusitis | COS | (Hopkins et al., 2018) | V | V | V | V | V | V | V | V | V | V | V | V | 12 |
| Hereditary Angioedema | COS | (Petersen et al., 2024) | V | V | V | V | V | V | V | 0 | V | V | V | 0 | 10 |
| COS, Core Outcome Set; HP, harmonisation process; V, standard met; O, unclear whether standard is met; X, standard not met; *NA, COS for clinical care and not for research. | | | | | | | | | | | | | | |  |

# Table S4 COS and Harmonisation processes currently under development.

| Condition | COS/HP | Initiative |
| --- | --- | --- |
| Atopic eczema | COS | (COMET Initiative \| A core outcome set for eczema treatment by Traditional Chinese medicine) |
|  |  | (COMET Initiative \| A core outcome set for atopic dermatitis treatment by Traditional Chinese medicine) |
|  |  | (Ronsch et al., 2020) |
| Asthma | COS | (Craig et al., 2020) |
|  |  | (COMET Initiative \| Developing a core outcome set of Traditional Chinese Medicine on Asthma) |
| Urticaria | COS | (COMET Initiative \| Developing a core outcome set for urticaria) |
| Allergic rhinitis | COS | (COMET Initiative \| Developing and validating the Core Outcome Set for Allergic Rhinitis in Traditional Chinese Medicine (COS-AR-TCM); COMET Initiative \| The Core Outcome Set for Adult’s Allergic Rhinitis with the Acupuncture and Moxibustion (COSAARAM) in Clinical Trials) |
|  |  | The Core Outcome Set for Adult’s Allergic Rhinitis with the Acupuncture and Moxibustion (COSAARAM) in Clinical Trials) |

COS, Core Outcome Set.

# Table S5 Methodology used in included HP and COS processes.

| Condition | COS/HP | Initiative | Outcome Identification | | Outcome prioritisation | | Instrument Selection method |
| --- | --- | --- | --- | --- | --- | --- | --- |
|  |  |  | Literature Review | Stakeholder Engagement | Delphi survey | Consensus Meeting |  |
|  |  |  | Systematic review, scoping review | Patient interviews, focus groups, surveys |  | Workshop, round table discussions etc. |  |
| Asthma | COS initiatives | (Sinha et al., 2012) | V | V | V | X | NA |
|  |  | (Tejwani et al., 2021) | V | V | V | V | NA |
|  |  | (Khaleva et al., 2023) | V | V | V | V | COSMIN methodology |
|  | Other harmonisation initiatives | (Reddel et al., 2009) | V | X | X | V | NA |
|  |  | (Busse et al., 2012) | V | X | X | V | NA |
|  |  | (Martínez-Moragón et al., 2024) | NA | NA | NA | NA | The process included systematic review, focus group with patients, Delphi and consensus meeting |
|  |  | (Gliklich et al., 2019) | V | X | X | V | NA |
| Eczema | COS initiatives | (Williams et al., 2022) | V | X | V | V | COSMIN methodology |
| Food allergy | COS initiatives | (Demidova et al., 2024) | X | V | V | V | NA |
|  |  | (COREOS  Collaborators et al., 2022) | V | V | V | V | As part of the Delphi together with outcomes |
| Coeliac disease. | Other harmonisation initiatives | (Ludvigsson et al., 2018) | V | X | X | V | NA |
| Urticaria | Other harmonisation initiatives | (Baiardini et al., 2011) | X | X | X | V | NA |
| Chronic Rhinosinusitis | COS initiatives | (Hopkins et al., 2018) | V | V | V | V | NA |
| Hereditary Angioedema | COS initiatives | (Petersen et al., 2024) | V | X | V | V | NA |

V = conducted; X, not conducted; NA, not applicable

# Table S6 Details of the stakeholders involved in the process.

| Initiative | Phase | Method | Participants (N) | Subgroups (as reported) | Geographical regions |
| --- | --- | --- | --- | --- | --- |
| Asthma | | | | | |
| (Sinha et al., 2012) [20] | Outcome Identification | Delphi | HCP/RS (46) | specialist respiratory paediatricians (16), general paediatricians (16), clinical academics (6), respiratory nurses (8) | Not specified |
|  |  |  | PR (49) | Parents of children with asthma | Not specified |
|  | Outcome voting | Delphi | HCP/RS (46) | specialist respiratory paediatricians, general paediatricians, clinical academics, respiratory nurses | Not specified |
|  |  |  | PR (50) | Parents of children with asthma | Not specified |
| (Tejwani et al., 2021) [21] | Outcome Identification | Key informant interviews | HCP/RS (4) | Clinicians/researchers | Not specified |
|  |  |  | PR (5) | Patient advocacy group | Not specified |
|  |  |  | Other (13) | Payer groups (3), HTA group (2), pharmaceutical companies (8) | Not specified |
|  | Outcome voting | 3 Delphi rounds and consensus meeting in between Delphi R2 and R3 | HCP/RS (12*) |  | Not specified |
|  |  |  | PR (13*) |  | Not specified |
|  |  |  | Other (20*) | Regulators (2), HTA group (8), industry (10) | Not specified |
| (Khaleva et al., 2023) [14] | Outcome Identification | Delphi 2 rounds | HCP/RS (29*) |  | Europe (29) |
|  |  |  | PR (11*) |  | Europe (10), North America (1) |
|  |  |  | Other (6*) | Pharmaceutical representatives (2), Health regulator (4) | Europe (5), North America (1) |
|  |  | Surveys to discuss priority outcomes | HCP/RS (27*) |  | Europe (27) |
|  |  |  | PR (14*) |  | Europe (13), North America (1) |
|  |  |  | Other (9*) | Pharmaceutical representatives (4), Health regulator (5) | Europe (8), North America (1) |
|  |  | Pan-European survey | PR (232) | Patient (201) Parent or carer (31) | Europe (232) |
|  | Outcome voting | Adult COMS meetings 3 rounds | HCP/RS (31*) |  | Europe (31) |
|  |  |  | PR (14*) |  | Europe (13), North America (1) |
|  |  |  | Other (9*) | Pharmaceutical representatives (4), Health regulators (5) | Europe (8), North America (1) |
|  |  | Pediatric COMS meetings 2 rounds | HCP/RS (36*) |  | Europe (36) |
|  |  |  | PR (13*) |  | Europe (12), North America (1) |
|  |  |  | Other (5*) | Pharmaceutical representatives (2), Health regulators (3) | Europe (4), North America (1) |
| (Reddel et al., 2009) | Outcome voting | Consensus workshop | Working group members (24) | | Not specified |
| (Busse et al., 2012) | Outcome voting | Expert subcommittee working group | 7 subcommittee group | | Not specified |
| (Martínez-Moragón et al., 2024) [16] | Outcome Identification | Focus group interviews | PR (4) |  | Not specified |
|  |  | 2 online Nominal group meeting | HCP/RS (19) | Hospital pharmacists (9), Pulmonologists (4), Allergologists (4), Nurses (2) | Spain (19) |
|  | Outcome voting | Delphi survey | PR (5) |  | Not specified |
|  |  |  | HCP/RS (58) | Hospital pharmacists (25), Pulmonologists (13), Allergologists (14), Nurses (11) | Not specified |
| (Gliklich et al., 2019) [12] | Outcome voting | Consensus meeting | Representatives of patient outcomes– focused registry, clinicians, researchers, representatives from medical specialty associations, health systems, community health centers, regulatory agencies, funding agencies, payers, patient advocacy organizations, measure developers, and measure-endorsement organizations | | Not specified |
| Eczema | | | | | |
| (Williams et al., 2022) [22] | Outcome voting | Delphi 3 rounds | HCP/RS (32*) |  | Australia (1), Europe (29), North America (11)  Other (5) |
|  |  |  | PR (6*) |  |  |
|  |  |  | Other (8*) | Journal editors (7), Regulatory agency representatives (1) |  |
|  | Instruments voting** | Consensus meeting  (HOME II) | HCP/RS (29) |  | Asia (8), Australia (1), Europe (30), North America (1), South America (3) |
|  |  |  | PR (5) |  |  |
|  |  |  | Other (9) | Methodologist (5), pharmaceutical industry representative (1), other (3) |  |
|  |  | Consensus meeting  (HOME III) | HCP/RS (33) |  | Asia (4), Europe (18), North America (32), South America (2) |
|  |  |  | PR (9) |  |  |
|  |  |  | Other (14) | Pharmaceutical industry representatives (7), Methodologist (5), Other (2) |  |
|  |  | Consensus meeting  (HOME IV) | HCP/RS (38) |  | Asia (7), Australia (2), Europe (48), North America (10), South America (3) |
|  |  |  | PR (12) |  |  |
|  |  |  | Other (20) | Methodologist (7), Pharmaceutical industry representative (13) |  |
|  |  | Consensus meeting  (HOME V) | HCP/RS (38) |  | Not specified |
|  |  |  | PR (13) |  |  |
|  |  |  | Other (29) | Industry representative (8), Independent (21) |  |
| IgE-mediated food allergy | | | | | |
| (Demidova et al., 2024)[11] | Outcome Identification | Hybrid Meeting | HCP/RS, PR |  |  |
|  | Outcome voting | Delphi 2 rounds | HCP/RS (332*) |  | Asia (32), Africa (4), Australia (8), Europe (532), North America (79), South America (10), Central America (5), Unknown (108) |
|  |  |  | PR (446*) |  |  |
|  |  |  | Others | Industry (non-voting) |  |
|  |  | Meeting participants | HCP/RS (24) |  | Asia (2), Europe (23), North America (5) |
|  |  |  | PR (6) |  |  |
|  |  |  | Others |  |  |
| EoE | | | | | |
| (Collaborators et al., 2022)[3] | Outcome Identification | Semi structured interviews | PR (36) | adult patients | Not specified |
|  |  | Survey | PR (154) | adult patients (109), Children and caregivers (49) | Not specified |
|  | Outcome voting | Delphi 2 rounds | HCP (69*) |  | Europe (24), North America (40), Other (5) |
|  |  | Consensus meeting | HCP (27) |  | Europe (8), North America (15), Other (4) |
| Coeliac disease | | | | | |
| (Ludvigsson et al., 2018)[15] | Outcome voting | Task force members | HCP/RS | Adult gastroenterologists (9), pediatricians (6), pathologist (1), basic scientists (1) | Representatives of 10 countries |
|  |  |  | PR (2) | representatives of patient organizations (2) |  |
| Urticaria | | | | | |
| (Baiardini et al., 2011)[1] | Outcome voting | Working group members |  |  | Not specified |
| Chronic Rhinosinusitis | | | | | |
| (Hopkins et al., 2018)[13] | Outcome Identification | Survey | HCP/RS (155) | ENT surgeons (116), respiratory and allergy specialists (11), GPs (8), nurses (3) | UK |
|  |  |  | PR (80) |  | Not specified |
|  |  | Patients focus group | PR (10) |  | Not specified |
|  | Outcome voting | Delphi | HCP/RS (88) | ENT specialists, Allergists, Respiratory physicians and primary care physicians | Not specified |
|  |  |  | PR (19) |  | Not specified |
| Hereditary Angioedema | | | | | |
| (Petersen et al., 2024)[17] | Outcome voting | Delphi | HCP/RS (42) |  | Representatives of 23 countries |
|  |  |  | PR (12) |  |  |
|  |  |  | Other (4) | Industry (3), Regulators (1) |  |
| HCP/RS – healthcare professionals/researchers; PR – patient representatives.  * The maximum number of participants throughout Delphi process is outlined ** Only meetings where voting for the instruments for clinical trial COS took place are included in the table | | | | | |
|  | | | | | |

# Table S7. Grouping individual outcomes into predefined domains across COS and harmonisation initiatives for clinical trials in allergic and immunologic conditions.

| Category | Initial outcome name | Initiative name |
| --- | --- | --- |
| Quality of life | Quality of life | (Sinha et al., 2012), (Reddel et al., 2009), (Busse et al., 2012) (Williams et al., 2022) (Demidova et al., 2024) (Ma et al., 2022) (Baiardini et al., 2011) |
|  | Patient-reported symptoms and QoL | (Hopkins et al., 2018) |
|  | Health-related Quality of life | Ludvigsson et al., 2018) |
|  | Asthma-specific quality of life | (Tejwani et al., 2021) (Khaleva et al., 2023) |
| Signs and symptoms | Allergic symptoms | (Demidova et al., 2024) |
|  | Symptoms | (Sinha et al., 2012), (Busse et al., 2012) (Baiardini et al., 2011) |
|  | Clinician-reported signs | (Williams et al., 2022) |
|  | Patient-reported symptoms | (Williams et al., 2022) (Ma et al., 2022) |
|  | Patient-reported symptoms and QoL | (Hopkins et al., 2018) |
|  | Clinical outcome assessment (including patient-reported outcomes) | Ludvigsson et al., 2018) |
|  | Need for rescue medication during entire the entire attack | (Petersen et al., 2024) |
|  | Symptom-free days | (Reddel et al., 2009) |
|  | Time to end of progression of all symptoms | (Petersen et al., 2024) |
|  | Reliever use | (Reddel et al., 2009) |
| Disease control | Composite scores | (Reddel et al., 2009) (Busse et al., 2012) |
|  | Change in asthma control | (Tejwani et al., 2021) |
|  | Change in overall symptom severity at one predetermined point between 15 min and 4 h after treatment | (Petersen et al., 2024) |
|  | Asthma control | (Khaleva et al., 2023) |
|  | Control of disease | (Hopkins et al., 2018) |
|  | Long-term control | (Williams et al., 2022) |
| Disease specific outcomes | Endoscopy | (Ma et al., 2022) |
|  | Histology | (Ma et al., 2022) |
|  | Lung function and related measurements | (Khaleva et al., 2023) |
|  | Post-BD FEV1 (for assessment of lung function decline) | (Reddel et al., 2009) |
|  | Pre-BD FEV1 (as predictor for exacerbations) | (Reddel et al., 2009) |
|  | Pulmonary physiology | (Busse et al., 2012) |
|  | Serology | Ludvigsson et al., 2018) |
| Exacerbations | Exacerbation (within last 1-4 wks.) | (Reddel et al., 2009) |
|  | Exacerbations | (Sinha et al., 2012) (Busse et al., 2012) |
|  | Severe asthma exacerbation | (Tejwani et al., 2021) |
|  | Healthcare utilization ( Severe exacerbations) | (Khaleva et al., 2023) |
| Healthcare utilisation | Asthma-specific emergency department visit | (Tejwani et al., 2021) |
|  | Asthma-specific hospital stay or admission | (Tejwani et al., 2021) |
|  | Healthcare utilisation and costs | (Busse et al., 2012) |
|  | Healthcare utilization ( Maintenance of oral corticosteroid use ) | (Khaleva et al., 2023) |
| Impact on daily activity | Impact on daily activity | (Hopkins et al., 2018) |
|  | Impairment of daily activities | (Petersen et al., 2024) |
| Treatment satisfaction | Treatment satisfaction | (Petersen et al., 2024) |
|  | Acceptability of treatment and side effects | (Hopkins et al., 2018) |
| Treatment side-effects | Acceptability of treatment and side effects | (Hopkins et al., 2018) |
|  | Treatment side-effects | (Reddel et al., 2009) |
| Mortality | Mortality | (Sinha et al., 2012) |
| Biomarkers | Biomarkers | (Busse et al., 2012) |

# References

1. Baiardini, I., Braido, F., Bindslev-Jensen, C., Bousquet, P. J., Brzoza, Z., Canonica, G. W., Compalati, E., Fiocchi, A., Fokkens, W., Gerth van Wijk, R., Gimenez-Arnau, A., Godse, K., Grattan, C., Grob, J. J., La Grutta, S., Kalogeromitros, D., Kocaturk, E., Lombardi, C., Mota-Pinto, A.,…Maurer, M. (2011). Recommendations for assessing patient-reported outcomes and health-related quality of life in patients with urticaria: a GA(2) LEN taskforce position paper. *Allergy*, *66*(7), 840-844. https://doi.org/10.1111/j.1398-9995.2011.02580.x
2. Busse, W. W., Morgan, W. J., Taggart, V., & Togias, A. (2012). Asthma outcomes workshop: overview. *J Allergy Clin Immunol*, *129*(3 Suppl), S1-8. https://doi.org/10.1016/j.jaci.2011.12.985
3. Collaborators, C., Ma, C., Schoepfer, A. M., Dellon, E. S., Bredenoord, A. J., Chehade, M., Collins, M. H., Feagan, B. G., Furuta, G. T., Gupta, S. K., Hirano, I., Jairath, V., Katzka, D. A., Pai, R. K., Rothenberg, M. E., Straumann, A., Aceves, S. S., Alexander, J. A., Arva, N. C.,…Safroneeva, E. (2022). Development of a core outcome set for therapeutic studies in eosinophilic esophagitis (COREOS). *J Allergy Clin Immunol*, *149*(2), 659-670. https://doi.org/10.1016/j.jaci.2021.07.001
4. *COMET Initiative | A core outcome set for atopic dermatitis treatment by Traditional Chinese medicine*. https://comet-initiative.org/Studies/Details/1990
5. *COMET Initiative | A core outcome set for eczema treatment by Traditional Chinese medicine*. https://www.comet-initiative.org/Studies/Details/1988
6. *COMET Initiative | Developing a core outcome set for urticaria*. https://comet-initiative.org/Studies/Details/3374
7. *COMET Initiative | Developing a core outcome set of Traditional Chinese Medicine on Asthma*. https://www.comet-initiative.org/Studies/Details/3050
8. *COMET Initiative | Developing and validating the Core Outcome Set for Allergic Rhinitis in Traditional Chinese Medicine (COS-AR-TCM)*. https://www.comet-initiative.org/Studies/Details/2575
9. *COMET Initiative | The Core Outcome Set for Adult’s Allergic Rhinitis with the Acupuncture and Moxibustion (COSAARAM) in Clinical Trials*. https://www.comet-initiative.org/Studies/Details/1504
10. Craig, S., Babl, F. E., Dalziel, S. R., Gray, C., Powell, C., Al Ansari, K., Lyttle, M. D., Roland, D., Benito, J., Velasco, R., Hoeffe, J., Moldovan, D., Thompson, G., Schuh, S., Zorc, J. J., Kwok, M., Mahajan, P., Johnson, M. D., Sapien, R.,…Pediatric Emergency Research, N. (2020). Acute severe paediatric asthma: study protocol for the development of a core outcome set, a Pediatric Emergency Reserarch Networks (PERN) study. *Trials*, *21*(1), 72. https://doi.org/10.1186/s13063-019-3785-6
11. Demidova, A., Drewitz, K. P., Kimkool, P., Banjanin, N., Barzylovich, V., Botjes, E., Capper, I., Castor, M. A. R., Comberiati, P., Cook, E. E., Costa, J., Chu, D. K., Epstein, M. M., Galvin, A. D., Giovannini, M., Girard, F., Golding, M. A., Greenhawt, M., Ierodiakonou, D.,…Consortium, C. (2024). Core Outcome Set for IgE-mediated food allergy clinical trials and observational studies of interventions: International Delphi consensus study 'COMFA'. *Allergy*, *79*(4), 977-989. https://doi.org/10.1111/all.16023
12. Gliklich, R. E., Castro, M., Leavy, M. B., Press, V. G., Barochia, A., Carroll, C. L., Harris, J., Rittner, S. S., Freishtat, R., Panettieri, R. A., Jr., & Mosnaim, G. S. (2019). Harmonized outcome measures for use in asthma patient registries and clinical practice. *J Allergy Clin Immunol*, *144*(3), 671-681 e671. https://doi.org/10.1016/j.jaci.2019.02.025
13. Hopkins, C., Hettige, R., Soni-Jaiswal, A., Lakhani, R., Carrie, S., Cervin, A., Douglas, R., Fokkens, W. J., Harvey, R., Hellings, P. W., Leunig, A., Lund, V. J., Philpott, C., Smith, T., Wang, D. Y., & Rudmik, L. (2018). CHronic Rhinosinusitis Outcome MEasures (CHROME), developing a core outcome set for trials of interventions in chronic rhinosinusitis. *Rhinology*, *56*(1), 22-32. https://doi.org/10.4193/Rhin17.247
14. Khaleva, E., Rattu, A., Brightling, C., Bush, A., Bossios, A., Bourdin, A., Chung, K. F., Chaudhuri, R., Coleman, C., Dahlen, S. E., Djukanovic, R., Deschildre, A., Fleming, L., Fowler, S. J., Gupta, A., Hamelmann, E., Hashimoto, S., Hedlin, G., Koppelman, G. H.,…Consortium, C. W. G. i. t. T. (2023). Development of Core Outcome Measures sets for paediatric and adult Severe Asthma (COMSA). *Eur Respir J*, *61*(4). https://doi.org/10.1183/13993003.00606-2022
15. Ludvigsson, J. F., Ciacci, C., Green, P. H., Kaukinen, K., Korponay-Szabo, I. R., Kurppa, K., Murray, J. A., Lundin, K. E. A., Maki, M. J., Popp, A., Reilly, N. R., Rodriguez-Herrera, A., Sanders, D. S., Schuppan, D., Sleet, S., Taavela, J., Voorhees, K., Walker, M. M., & Leffler, D. A. (2018). Outcome measures in coeliac disease trials: the Tampere recommendations. *Gut*, *67*(8), 1410-1424. https://doi.org/10.1136/gutjnl-2017-314853
16. Martínez-Moragón, E., Ignacio, A. E., María, M. G., Francisco, C. M., Marta, C. L., Eusebi, C. V., Carlos, C. D., David, D.-P., Ibon, E. G., Sara, G. G., Ruperto, G.-P., Alicia, H. M., Tamara, H. V., Sònia, J. M., Valentín, L.-C., Icíar, M. L., Vicente, M.-B., Álvaro, M.-A., Cristina, M. C. A.,…and Sánchez-Cuellar, S. (2024). Patient-reported outcome measures in severe asthma: an expert consensus. *Journal of Asthma*, *61*(6), 619-631. https://doi.org/10.1080/02770903.2023.2297372
17. Petersen, R. S., Fijen, L. M., Apfelbacher, C., Magerl, M., Weller, K., Aberer, W., Adatia, A., Audhya, P., Bara, N. A., Betschel, S., Boccon-Gibod, I., Bouillet, L., Brodszki, N., Busse, P. J., Buttgereit, T., Bygum, A., Cancian, M., Craig, T., Csuka, D.,…Cohn, D. M. (2024). A Core Outcome Set for Efficacy of Acute Treatment of Hereditary Angioedema. *J Allergy Clin Immunol Pract*, *12*(6), 1614-1621. https://doi.org/10.1016/j.jaip.2024.04.007
18. Reddel, H. K., Taylor, D. R., Bateman, E. D., Boulet, L. P., Boushey, H. A., Busse, W. W., Casale, T. B., Chanez, P., Enright, P. L., Gibson, P. G., de Jongste, J. C., Kerstjens, H. A., Lazarus, S. C., Levy, M. L., O'Byrne, P. M., Partridge, M. R., Pavord, I. D., Sears, M. R., Sterk, P. J.,…Exacerbations. (2009). An official American Thoracic Society/European Respiratory Society statement: asthma control and exacerbations: standardizing endpoints for clinical asthma trials and clinical practice. *Am J Respir Crit Care Med*, *180*(1), 59-99. https://doi.org/10.1164/rccm.200801-060ST
19. Ronsch, H., Apfelbacher, C., Brans, R., Ofenloch, R., Schuttelaar, M. L. A., Weisshaar, E., & Bauer, A. (2020). Protocol for the development of a core domain set for hand eczema trials. *J Eur Acad Dermatol Venereol*, *34*(12), 2871-2876. https://doi.org/10.1111/jdv.16429
20. Sinha, I. P., Gallagher, R., Williamson, P. R., & Smyth, R. L. (2012). Development of a core outcome set for clinical trials in childhood asthma: a survey of clinicians, parents, and young people. *Trials*, *13*, 103. https://doi.org/10.1186/1745-6215-13-103
21. Tejwani, V., Chang, H. Y., Tran, A. P., Naber, J. A., Gutzwiller, F. S., Winders, T. A., Khurana, S., Sumino, K., Mosnaim, G., Moloney, R. M., & core, A. p. (2021). A multistakeholder Delphi consensus core outcome set for clinical trials in moderate-to-severe asthma (coreASTHMA). *Ann Allergy Asthma Immunol*, *127*(1), 116-122 e117. https://doi.org/10.1016/j.anai.2021.03.022
22. Williams, H. C., Schmitt, J., Thomas, K. S., Spuls, P. I., Simpson, E. L., Apfelbacher, C. J., Chalmers, J. R., Furue, M., Katoh, N., Gerbens, L. A. A., Leshem, Y. A., Howells, L., Singh, J. A., Boers, M., & Initiative, H. (2022). The HOME Core outcome set for clinical trials of atopic dermatitis. *J Allergy Clin Immunol*, *149*(6), 1899-1911. https://doi.org/10.1016/j.jaci.2022.03.017
